# Polymerization retardation isothermal amplification (PRIA): a strategy enables sensitively quantify genome-wide 5-methylcytosine oxides rapidly on handy instruments with nanoscale sample input

**DOI:** 10.1093/nar/gkz704

**Published:** 2019-08-16

**Authors:** Danping Chen, Yang Wang, Mingming Mo, Junjie Zhang, Yanfei Zhang, Yuzhi Xu, Si-Yang Liu, Jun Chen, Yingjun Ma, Li Zhang, Zong Dai, Chun Cai, Xiaoyong Zou

**Affiliations:** 1 School of Chemistry, Sun Yat-Sen University, Guangzhou 510275, China; 2 Guangdong Key Laboratory for the Research and Development of Natural Drugs, Guangdong Medical College, Zhanjiang, Guangdong 524023, China

## Abstract

The current methods for quantifying genome-wide 5-methylcytosine (5mC) oxides are still scarce, mostly restricted with two limitations: assay sensitivity is seriously compromised with cost, assay time and sample input; epigenetic information is irreproducible during polymerase chain reaction (PCR) amplification without bisulfite pretreatment. Here, we propose a novel Polymerization Retardation Isothermal Amplification (PRIA) strategy to directly amplify the minute differences between epigenetic bases and others by arranging DNA polymerase to repetitively pass large electron-withdrawing groups tagged 5mC-oxides. We demonstrate that low abundant 5-hydroxymethylcytosine (5hmC), 5-formylcytosine (5fC) and 5-carboxycytosine (5caC) in genomic DNA can be accurately quantified within 10 h with 100 ng sample input on a laboratory real-time quantitative PCR instrument, and even multiple samples can be analyzed simultaneously in microplates. The global levels of 5hmC and 5fC in mouse and human brain tissues, rat hippocampal neuronal tissue, mouse kidney tissue and mouse embryonic stem cells were quantified and the observations not only confirm the widespread presence of 5hmC and 5fC but also indicate their significant variation in different tissues and cells. The strategy is easily performed in almost all research and medical laboratories, and would provide the potential capability to other candidate modifications in nucleotides.

## INTRODUCTION

DNA methylation is a complex process that is dynamic and reversible. Facilitated by ten-eleven translocation (Tet) proteins, 5-methylcytosine (5mC) is oxidized stepwise to 5-hydroxymethylcytosine (5hmC), 5-formylcytosine (5fC) and 5-carboxycytosine (5caC), and can be further converted back to cytosine (C) from 5fC and 5caC with the aid of thymine-DNA-glycosylase (Tdg) (1–4). This pivotal process can reset epigenetic information and gene transcription, thus plays important roles in many physiological functions including cell maintenance and differentiation (5), gene regulation (6), DNA structures alterations (7), etc. Quantifying the global levels of these 5mC-oxides in genome-wide is more practical for providing valuable epigenetic information in deciphering physiological mechanisms and identifying the initiation of disease in a relatively fast and low-cost way.

Despite a decade of dedicated studies, the methods for quantifying these bases are still scarce because the abundances of 5mC-oxides in genomes are extremely low and the differences among their structures are subtle. The global level of 5hmC in a genome is almost 10-fold lower than that of 5mC (8,9), the abundance of 5fC is yet 100-fold lower than 5mC (10,11), and the 5caC content is even less that only ∼3.0 × 10^–6^ in C can be found in the genome of a mouse embryonic stem cell (mESC) (1,12,13). MS-based methods, particularly liquid chromatography tandem mass spectrometry (LC-MS/MS) (14) and ultrahigh performance liquid chromatography-mass spectrometry coupled with multiple-reaction monitoring (UHPLC-MRM-MS/MS) (15,16), are currently the most powerful tools for analysis of 5mC-oxides. Oligonucleotides (oligos) are digested into nucleotides, and the different cytosine forms are separated by chromatography and quantified by MS. In addition to sophisticated and expensive instruments, sample enrichment and chemical derivatization are commonly required, resulting in long analytical times (longer than 24 h) and high sample requirements (1–4 μg) (8,10,17,18). The other prevalent strategies are polymerase chain reaction (PCR)-based methods, in which the DNA methylation status is determined by sequencing of PCR products. As 5mC-oxides do not interfere with Watson–Crick base pairing, epigenetic information will lost in duplicate strands during PCR process. Therefore, the current strategies mostly have to rely on bisulfite treatment to transfer the epigenetic information into sequences difference (19,20). For instance, a quantitative method termed reduced bisulfite sequencing (redBS-Seq) has been developed to profile 5fC in genomic DNA. The 5fC in genome is selectively reduced to 5hmC by NaHB_4_, followed by bisulfite treatment (21). Similarly, an oxidative bisulfite sequencing (oxBS-Seq) method has been proposed for mapping 5hmC in genome by selective chemical oxidation of 5hmC to 5fC enables bisulfite conversion of 5fC to uracil (22). Although these methods can provide sufficient sensitivity and even detailed site information with single-base resolution, they are even more costly and time-consuming (about 40–50 h), and require a large amount of samples (1–3 μg). Moreover, bisulfite treatment is harsh and time-consuming, and likely to damage DNA, resulting in false-positives or negatives (23,24). Few research has been reported to overcome the limitations of bisulfite treatment. 3-azido-N-(2-(cyanomethyl)benzo[d] thiazol-6-yl)propanamide (azi-BP) was found able to block the exocyclic 4-amino group of 5fC, leading to mismatch and subsequent sequence difference after PCR process (25). The 5fC level at specific-loci of genome was analyzed without bisulfite treatment, whereas, the reaction between 5fC and azi-BP requires a relatively high temperature (56°C) and a long reaction time (18 h).

Though the present methods for 5mC-oxides analysis can provide epigenetic information to a certain extent, clear limitations are still involved. (i) Methods mostly rely on sophisticated instruments and must compromise among sensitivity, cost, speed and sample input. (ii) Epigenetic information is irreproducible, thus can be barely amplified directly by PCR without bisulfite treatment. Expensive and time-consuming approaches that do not rapidly allow the elucidation of DNA methylation have greatly restricted their wide applications, especially since epigenetic bases are likely to be recognized as disease markers in the future.

It is known that DNA polymerase exhibits different extension rates on duplicate strands as passing disparate bases (26,27), providing the possibility of differentiating different bases during polymerization. A preliminary method was proposed recently, using a supramolecular aldehyde reactive probe to prevent DNA polymerase from binding to 5fC site (28). The 5fC site was distinguished because of the inhibition of the PCR process on the 5fC-oligos, whereas the genome-wide 5fC content was not quantified. Herein, we propose a strategy, Polymerization Retardation Isothermal Amplification (PRIA), enabling robust, rapid, and cost-effective quantitation of the global levels of 5fC, 5hmC and 5caC in low-input genomes on a handy instrument. After genomic 5fC, 5hmC and 5caC are respectively tagged with *p*-hydrazinobenzenesulfonic acid (PHPA), 3-carboxyphenyl-boroic acid (3-CPBA) and lucifer yellow CH dipotassium salt (LY), DNA polymerase is arranged to pass the tagged epigenetic bases repetitively and consecutively on every genomic fragment through isothermal replication-scission amplification reaction. Information for low abundant 5fC, 5hmC and 5caC in genomic DNA is amplified conveniently on a laboratory real-time quantitative PCR (RT-qPCR) instrument, reflecting in a clear decrease in the efficiency of polymerization. We demonstrate that the tagging reactions are highly specific and efficient, and can greatly retard polymerase extension. The PRIA strategy can sensitively quantify the global levels of 5mC-oxies with greatly reduce assay time and sample requirement. The global levels of 5hmC and 5fC in mouse and human brain tissues, rat hippocampal neuronal tissue, mouse kidney tissue and mESCs were accurately quantified, and the observations not only confirm the widespread presence of 5hmC and 5fC but also indicate their significant variation in different tissues and cells.

## MATERIALS AND METHODS

### Materials

Oligos were purchased from TaKaRa (Dalian, China) and purified by HPLC before use. The sequences of the oligos used are listed in Supplementary Table S1. Human brain DNA were purchased from Epigentek (USA).

5mdC, 5hmdC, 5fdC and 5cadC were obtained from Active-Motif (Carlsbad, CA, US). Klenow fragment polymerase (KF, 3′ → 5′ exo-), Nt.BsmAI NEase, T4 Phage *β*-glucosyltransferase (T4-*β*GT), UDP-glucose, Cutsmart buffer (20 mM Tris-acetate, 50 mM potassium acetate, 10 mM magnesium acetate and 0.1 mg ml^–1^ BSA, pH 7.9), NEB buffer 2 (10 mM Tris–HCl, 10 mM MgCl_2_, 50 mM NaCl and 1 mM dithiothreitol, pH 7.9), NEB buffer 4 (50 mM potassium acetate, 20 mM Tris-acetate, 10 mM magnesium acetate and 1 mM dithiothreitol, pH 7.9), the NEBNext Ultra End Repair/dA-Tailing Module, the NEBNext Ultra Ligation Module and NEBNext Singleplex Oligos for Illumina were purchased from New England Biolabs (USA). Deoxynucleotide triphosphates (dNTPs), UNIQ-10 Spin Column Oligo DNA Purification Kits, EZ-10 Column DNA Purification Kits and 1 × phosphate buffer saline (PBS, pH 7.4) were ordered from Shanghai Sangon Biological Engineering Technology Services Co., Ltd. (Shanghai, China). PHPA, 3-CPBA, LY and morpholine ethylsulfonic acid were purchased from Aladdin and used without further purification (Shanghai, China). Acrylamide/bis-acrylamide 40% solution, ammonium peroxodisulfate, tetramethylethylenediamine, biotin hydrazide, 1-ethyl-3-[3-dimethylaminopropyl]-carbodiimide hydrochloride (EDC) and *N*-hydroxysuccinimide (NHS) were obtained from Sigma-Aldrich (USA). SYBR Green II was obtained from Xiamen Bio-Vision Biotech. Co. Ltd. (Xiamen, China). Other regents of analytical grade were obtained from Beijing Chemical Co. (Beijing, China) and used without further purification.

### Principle of PRIA strategy

**Figure 1.**
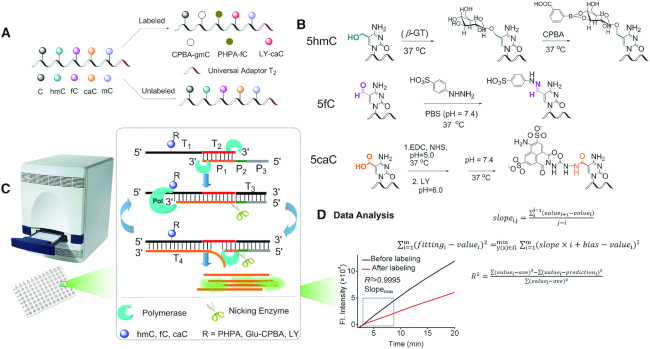
Principle of PRIA assay for genome-wide 5hmC, 5fC and 5caC detection. (**A**) Universal adaptor T_2_ ligated genomic ssDNA fragments with or without labeling. (**B**) Chemical labeling of 5fC-DNA, 5hmC-DNA and 5caC-DNA by PHPA, T4-*β*GT/UDP-glucose and 3-CPBA, and LY, respectively. (**C**) Isothermal replication-scission amplification reaction of the ssDNA fragments on a laboratory rt-qPCR instrument. (**D**) Data analysis for screening credible slope.

The basic idea of the PRIA strategy is to amplify the minute differences between the epigenetic bases and others by forcing DNA polymerase to pass the target sequences repetitively. The assay of the PRIA strategy involves four main procedures, including sample fragmentation and adaption, epigenetic loci tagging, polymerase screening and data analysis (Figure 1). Genomic double-stranded DNA (dsDNA) is extracted from the biological samples, sheared into small fragments and then end-repaired for ligation of the universal adaptor T_2_. After being denatured into single-stranded DNA (ssDNA), the T_2_ adapted fragments are divided equally into A and B parts: the A part is for epigenetic loci tagging, and the B part is left for control. Molecules with large strong electron-withdrawing groups are specifically introduced to the 5fC, 5hmC and 5caC, respectively, enabling distinguish target epigenetic bases from other bases and improve sensitivity by further retarding polymerase extension. 5fC is introduced with a benzenesulfonic acid group to its formyl group by the reaction with PHPA. 5hmC is firstly converted to 5gmC specifically via transferring glucose to the hydroxyl in 5hmC by T4-*β*GT and UDP-glucose (29,30). The *cis*-diol groups in the glucose moiety of 5gmC-DNA are capable for immobilizing 3-CPBA through cyclization reaction, forming CPBA-gmC-DNA. For 5caC, LY with large steric hindrance and hydrazine group is feasibly tagged to the activated carboxyl in 5caC by amidation reaction. The A and B parts are respectively mixed with primer, DNA polymerase and nicking enzyme for isothermal replication-scission amplification reaction, allowing the ssDNA fragments being screened repetitively by DNA polymerase. The primer has a hybrid sequence P_1_ (orange part) that is fully complementary to the universal adaptor T_2_, a nicking enzyme recognition site P_2_ (green part), and an extension sequence P_3_ (gray part) that is long enough to ensure that the primer stably hybridizes with target DNA fragments for the polymerization process to occur at the assay temperature. When the primer hybridizes to the target fragments with T_2_, extension from its 3′ terminus begins along with the target fragment T_1_, allowing DNA polymerase to pass the tagged epigenetic loci one time. The weak discrepancy between the tagged and untagged bases accumulates one time. Meanwhile, extension of the target fragment from its 3′ terminus along with P_2_ and P_3_ of the primer as template results in a nicking enzyme recognition site. The nicking enzyme cleaves at the next base downstream of the recognition site in the formed dsDNA, creating a 3′ terminus that enables the next round of DNA extension. In each DNA extension process, DNA polymerase passes the tagged epigenetic loci one time, producing one T_4_ ssDNA. As the isothermal replication-scission amplification reactions are consecutively and constantly repeated for every target DNA fragment, all the tagged epigenetic loci are screened by DNA polymerase repetitively. Extremely lowly abundant levels—even one site of 5hmC, 5fC or 5caC in genomic DNA—can be amplified and detected as a clear decrease in polymerization efficiency. As a result, the amount of T_4_ differs increasingly with reaction time, which is reflected in the clearly different slopes of the linearly increasing fluorescence signal of the fluorescent dye (SYBR Green II). The credible slope is assured with data processing by screening a maximal slope in the confidence interval with *R*^2^ (the fitting degree of the regression line; range of 0–1) >0.9995. The global levels of 5hmC, 5fC and 5caC in genomic DNA can be evaluated by determining the difference between the slope of the A part and that of an equal amount of the B part (Δ*S*, also the polymerization efficiency difference). The PRIA strategy would have several significant strengths. The assay can be performed isothermally in microplates on laboratory handy instruments such as an RT-qPCR instrument or a microplate reader. The chemical tagging reactions can be performed under mild conditions in one or two-step reactions, avoiding DNA damage and reducing sample input and analytical time. The PRIA strategy is promising in greatly facile the epigenetic researches.

### Test DNAs for proof-of-concept

#### Epigenetic bases labeling

For test 5fC-DNA, 50 mM PHPA and 2 μM test 5fC-DNA were incubated at 37°C for 6 h in the presence of 100 mM PBS buffer (pH 7.4).

For test 5hmC-DNA, 40 μM UDP-glucose, 2 μM test 5hmC-DNA, 1 × NEB buffer 4 and 0.1 U of T4-*β*GT were incubated at 37°C for 2 h. A mixture of freshly prepared 1.8 μM gmC-DNA and 50 mM 3-CPBA (dissolved in 200 mM Na_2_HPO_4_ buffer) were incubated at 37°C for 6 h.

For test 5caC-DNA. 2 μM test 5caC-DNA, 2 mM EDC, 0.4 mM NHS and 75 mM MES buffer (pH 5.0) were incubated at 37°C for 1 h. Subsequently, 200 μM LY and 75 mM MES buffer (pH 6.0) were added, and the reaction was continued at 37°C for 30 min at which time 150 mM NaCl and PBS buffer (pH 7.4) were added to the reaction and incubated at 37°C for 8 h.

After each labeling reaction, ssDNA was purified using Oligo DNA Purification Kits to remove chemicals and enzymes. The labeling products were verified by MALDI-TOF MS ultrafleXtreme (Bruker, Germany), and 3-hydroxypicolinic acid (3-HPA) as the assisted matrix. Their concentrations were evaluated via UV absorbance at 260 nm on a Nanodrop 2000c spectrophotometer. The melting temperature (*T*_m_) values of test dsDNAs before and after labeling were evaluated from their melting curves. The melting curves were determined on a CFX Connect Real-Time System (Bio-Rad, USA) by recording the fluorescence signal changes that occurred with the increase in the temperature from 45 to 90°C at a rate of 5°C min^−1^.

#### Isothermal replication-scission amplification reaction

The conditions for amplification reactions were firstly optimized, including the concentration of template DNA, the content of primer, the concentrations of KF polymerase and Nt.BsmAI NEase, respectively.

After the optimization of the reaction conditions, the amplification reactions for the proof-of-concept study were conducted at 37°C for 60 min in 20 μl solutions containing 100 nM amplification templates (5′-GAG ACC GGA GTC CGC TTT CCT CTT C**X**G GAA AAT GTA AGC CGA ACC TAA AGC AAT CAC CAG GG-3′; **X** is C, 5mC, 5hmC, 5fC, 5caC, respectively, Supplementary Table S1), 20 nM primer, 0.05 U of KF polymerase, 0.5 U of Nt.BsmAI NEase, 1 × NEB buffer 2, 1 × Cutsmart buffer, dNTPs and SYBR Green II. Fluorescence signals were recorded every min using an RT-qPCR instrument.

### Quantification of the genome-wide 5-methylcytosine oxides in genomic DNAs

#### Genomic DNA extraction

Genomic DNAs from mESCs, new-born and adult mouse brain tissues, adult mouse kidney tissue, and rat hippocampal neuronal tissue were extracted with Biospin Cell Genomic DNA Extraction Kits and Biospin Tissue Genomic DNA Extraction Kits (Hangzhou Bioer Co. Ltd.) according to the manufacturer's instructions. The extracted DNAs were quantified via UV absorbance at 260 nm on a Nanodrop 2000c spectrophotometer and stored at −20°C.

#### Sample fragmentation

Genomic DNA was sheared into ∼200-bp fragments on a Covaris™ M220 Focused-ultrasonicator™ Instrument with the peak incident power set to 50 W, a duty factor of 20 and 200 cycles per burst at 20°C for 160 s. The sample fragments were characterized by 8% PAGE at 140 V for 40 min at room temperature, stored at −20°C before use.

#### Adaptor ligation

The maximal amount of input material during PRIA is the adaptor ligation step. Based on the manufacturer's instruction, 100 ng of the DNA fragments were repaired by reacting them with the End Prep Enzyme Mix and End Repair Reaction Buffer at 20°C for 30 min and then at 65°C for 30 min. Subsequently, they were reacted with the Blunt/TA Ligase Master Mix, the NEBNext Adaptor for Illumina and Ligation Enhancer at 20°C for 15 min before finally being treated with the USER™ enzyme at 37°C for 15 min. The adaptor-containing fragments were labeled with PHPA to obtain chemically labeled fragments, after which they were denatured at 95°C for 10 min and immediately transferred to ice to acquire single stranded DNA.

#### Labeling of epigenetic bases in genomic DNA fragments

The labeling of the epigenetic bases in genomic DNA fragments was performed following the labeling processes for test DNAs under the optimal conditions. After each labeling reaction, ssDNA was purified using EZ-10 Column DNA Purification Kits to remove chemicals and enzymes. Their concentrations were evaluated via UV absorbance at 260 nm on a Nanodrop 2000c spectrophotometer.

#### Isothermal replication-scission amplification reaction

The amplification reaction for biological samples was conducted at 37°C for 30 min in 20 μl solutions containing 32.8 nM target fragments (5hmC-DNA and CPBA-gmC-DNA, 5fC-DNA and PHPA-fC-DNA, 5caC-DNA and LY-caC-DNA, respectively), 50 nM primer, 0.1 U of KF polymerase, 0.5 U of Nt.BsmAI NEase, 1 × NEB buffer 2, 1 × Cutsmart buffer, dNTPs and SYBR Green II. Fluorescence signals were recorded every min using an RT-qPCR instrument.

#### Genome-wide 5hmC and 5fC quantification

Genome-wide detection of 5hmC in DNA was performed using an isotope-based LC-MS/MS method (31). For each sample, 1 μg of hydrolyzed genomic DNA sample was analyzed by LC-MS/MS on an Agilent 6430B triple quadrupole mass spectrometer with an Agilent 1200 series LC system (Agilent Technologies, CA, USA), The column temperature was set at 20°C. The eluting buffers contained 2.5 mM ammonium formate in milli-Q water and acetonitrile. Quantification was accomplished in SRM mode.

Genome-wide detection of 5fC in DNA was performed using a MethylFlash™ 5-Formylcytosine DNA Quantification Kit (Colorimetric, US Epigentek Group Inc) according to the manufacturer's instructions. Briefly, 300 ng of genomic DNA were bound to the assay plates at 37°C for 90 min; 5fC-DNA was captured by adding 50 μl of the capture antibody, followed by incubation at room temperature for 60 min; next, 50 μl of the detection antibody were added, with a 30 min incubation at room temperature; then 50 μl of enhancer solution was added as last step description. All of the above steps, washes with washing buffer and removed three times after each addition. At last, 50 μl of the developer solution was added, and after 10 min of incubation, 50 μl of the stop solution were added, followed by measurement of the absorbance on a microplate reader at 450 nm.

### Data processing

Data manipulation was performed in Python (version 3.5) with scipy (version 0.19.0) and numpy (version 1.11.3). All the experimental data from each real-time fluorescence spectrum were analyzed using the equation (1) to calculate all the possible slopes of the amplification plot.

(1) $$\begin{equation*}{\ slope}_{i,j} = \frac{{\sum\nolimits_i^{j - 1} {\left( {{\ value}_{i + 1} - {\ value}_i} \right)} }}{{j - i}}\end{equation*}$$

Where *slope*_i,j_ indicates the slope from the *i*^th^ cycle to the *j*^th^ cycle in the plot, and *value*_i_ is the fluorescence intensity of *i*^th^ cycle.

After calculating all the possible slopes of the amplification plot, error estimations for these slopes were performed using the equation (2).

(2) $$\begin{eqnarray*}&&\sum\nolimits_{i = 1}^m {\left({{\ fitting}_i - {\ value}_i} \right)}^2 \nonumber\\ &&\quad = {{}_{y\left( x \right) \in \Omega }^{\min }}\sum\nolimits_{i = 1}^m {{{\left( {{\ slope }\times i + {\ bias }- {\ value}_i} \right)}^2}} \end{eqnarray*}$$

Where *fitting*_i_ represents the linear fitting value at the *i*^th^ cycle, *m* represents amount of whole cycles, *slope* is a definite value in the designated scope, and *bias* minimizes the total mean square error of the linear fitting.

Ordered error estimations above *R*^2^ for each slope were manipulated using the equation (3).

(3) $$\begin{eqnarray*}{R^2} = \frac{{\sum {{{\left( {{\ value}_i - ave} \right)}^2} - \sum {{{\left( {{\ value}_i - {\ prediction}_i} \right)}^2}} } }}{{\sum {{{\left( {{\ value}_i - {\ ave}} \right)}^2}} }}\nonumber\\ \end{eqnarray*}$$

Where *ave* is the average value of all the *value*_i_, and *prediction*_i_ is derived from *slope* × *i* + *bias*. *R*^2^ > 0.999 was screened as a normative reference for the data analysis.

## RESULTS

### Proof-of-concept study

**Figure 2.**
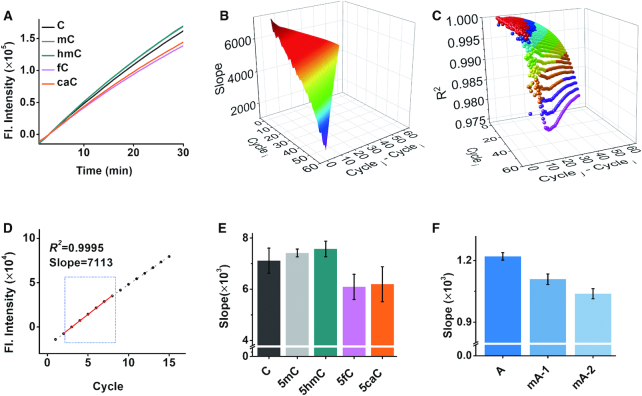
Effect of epigenetic modification on polymerization efficiency of polymerase. (**A**) Time-dependent fluorescence spectra of the isothermal replication-scission amplification reactions with C-DNA, 5mC-DNA, 5hmC-DNA, 5fC-DNA and 5caC-DNA. (**B**) Data processing for calculating all the slopes from the plot of the isothermal replication-scission amplification reaction with C-DNA, cycle_i_ presents the initial screening cycle site; cycle_j_ − cycle_i_ presents the interval. (**C**) Data processing for screening *R*^2^. (**D**) The screened confidence interval in the plot of the isothermal replication-scission amplification reaction with C-DNA. (**E**) Histogram of the slopes of the isothermal replication-scission amplification reactions with C-DNA, 5mC-DNA, 5hmC-DNA, 5fC-DNA and 5caC-DNA. (**F**) Histogram of the isothermal replication-scission amplification reaction slope with A-RNA, mA1-RNA and mA2-RNA. The conditions of the isothermal replication-scission amplification reaction were: 0.5 U of Nt.BsmAI NEase, 0.05 U of KF polymerase, 100 nM template and 20 nM primer.

First, we constructed a model to study whether DNA polymerase would exhibit disparate polymerization efficiency on different cytosine forms. For this proof-of-concept study, a part of the T53 MPRM10823 promoter with 62 nt was chosen as a template, together with KF polymerase and Nt.BsmAI NEase, for isothermal replication-scission amplification reaction under optimal conditions (Supplementary Figure S1). As expected, with increasing reaction time, the fluorescence intensity increased linearly with different slopes (Figure 2A), reflecting distinct polymerization efficiencies in the amplification reactions. To precisely analyze this difference, the plot data (fluorescence intensity versus sampling circle number) from C-DNA were input into the formulas for data processing using the Python language. All the possible linearities were fitted and their slopes and *R*^2^ were ordered from the maximum to the minimum (Figure 2B and C, as for example). The credible data was obtained from slope and *R*^2^ screening (Figure 2D and Supplementary Table S2). The polymerization efficiency was differentiated, showing an order of 5hmC-DNA ≥ 5mC-DNA > C-DNA > 5caC-DNA ≥ 5fC-DNA (Figure 2E). The polymerization efficiency of the KF polymerase on 5fC-DNA and 5caC-DNA was reduced by ∼14.4% compared to that on C-DNA.

Besides DNA, the changes of polymerization efficiency occur with other types of base modifications in variant oligos. *N*^6^-methyladenine (6mA), one of the prevalent modifications that occur in RNA, was also discriminated. The RNAs containing zero, one or two 6mA sites (A-RNA, mA1-RNA and mA2-RNA, respectively) were used as templates for the isothermal replication-scission amplification reaction. As shown in Figure 2F, compared to that on A-RNA, the polymerization efficiency of the KF polymerase decreased by ∼9.1% on the mA1-RNA and further decreased by ∼14.9% on mA2-RNA, demonstrating that the extension of DNA polymerase can be hindered by 6mA (32–34). The changes of polymerization efficiency accumulate for oligos with increasing amounts of base differences. Therefore, by comparatively studying the polymerization efficiency of DNA polymerase on target oligos, possible base changes in the target oligos can be promisingly differentiated and even quantified in a facile way.

### Feasibility of PRIA for detection of 5fC, 5hmC and 5caC

The presupposition of the PRIA strategy is that the 5mC-oxides can be specifically and efficiently labeled with large and distinctive molecules. These molecules were chosen to constitute a large inhibiting group and a linking group. According to the properties and spatial structure of DNA polymerase, the inhibiting group should have a strong electron-withdrawing inductive effect or provide large steric hindrance. Nitro, sulfonyl, carboxyl and tertiary butyl groups were considered.

**Figure 3.**
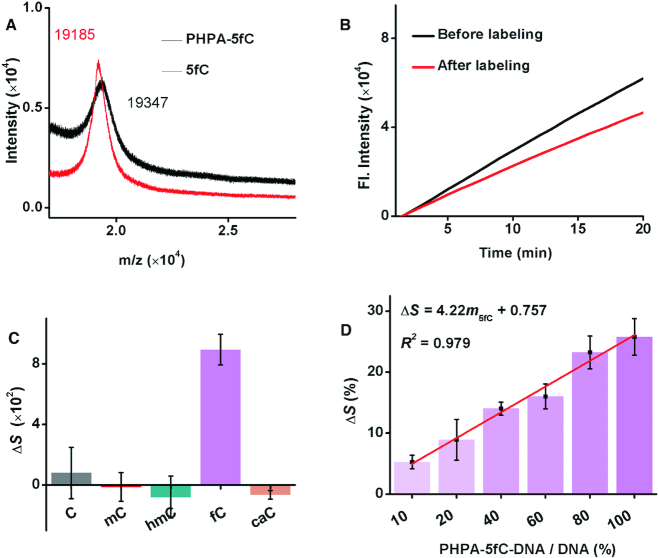
Feasibility of PRIA for the detection of 5fC. (**A**) MALDI-TOF MS characterization of 5fC-DNA before and after labeling with PHPA. (**B**) Time-dependent fluorescence spectra of the isothermal replication-scission amplification reactions with 5fC-DNA before and after labeling with PHPA. (**C**) Histogram of Δ*S* for the isothermal replication-scission amplification reactions with C-DNA, 5mC-DNA, 5hmC-DNA, 5fC-DNA and 5caC-DNA before and after their reactions with PHPA. (**D**) Histogram of Δ*S* for the isothermal replication-scission amplification reactions with diverse proportions of PHPA-5fC-DNA, and plot of Δ*S* versus PHPA-5fC-DNA content.

The aldehyde in 5fC is a relatively active group that can feasibly react with many nucleophiles, such as primary amines, hydroxylamine and hydrazine. In contrast to the unstable Schiff base produced by the reaction of an aldehyde with a primary amine, the hydrazone derivative produced from a hydrazine and an aldehyde is more stable under aqueous conditions. Therefore, water soluble PHPA, which has sulfonyl and hydrazine groups, was chosen as a potential tag for 5fC. After 5fC and PHPA reacted at 37°C and neutral pH (7.4) for 6 h, the main signal of PHPA-5fC-DNA was observed by MALDI-TOF MS at m/z 19347 (Figure 3A), and the *T*_m_ of the double-stranded 5fC-DNAs reduced by 1.5°C after reacting with PHPA (Supplementary Figure S2 and Table S3). Moreover, when PHPA was reacted with 5-methyl-2′-deoxycytidine (5mdC), 5-hydroxymethyl-2′-deoxycytidine (5hmdC), 5-formyl-2′-deoxycytidine (5fdC) and 5-carboxy-2′-deoxycytidine (5cadC) under the same conditions, LTQ-orbitrap-MS analysis showed that only 5fdC provided a distinct signal for PHPA-5fdC with m/z 424.07 (Supplementary Figure S3a); no detectable product signals were observed for PHPA-5mdC, PHPA-5hmdC and PHPA-5cadC (Supplementary Figure S3b–d). The results indicate that the labeling reaction can be performed rapidly and specifically. The sulfonic acid group in PHPA would ensure a sufficient reaction between 5fC-DNA and PHPA by enhancing its water solubility. Approximately 64.8% of 5fC-DNA was converted into PHPA-5fC-DNA after the reaction, as evaluated by HPLC (monitored at 260 and 345 nm) (Supplementary Figure S4 and Table S4). Besides, the labeling efficiency almost kept constant on various DNAs samples. As verified by performing labeling reactions on different DNA mixtures, little discrepancy of polymerization efficiency (the slopes) was observed from different tagged 5fC samples (Supplementary Figure S5a and b), suggesting that the labeling efficiency did not vary among samples.

Next, we verified that the PHPA adduct would achieve quantitative information for 5fC. After being purified with a DNA Purification Kit to remove organic and salt impurities, the PHPA-5fC-DNA was subjected to the isothermal replication-scission amplification reaction. The polymerization efficiency of the KF polymerase on PHPA-5fC-DNA was pronouncedly reduced by ∼25.8% of that on 5fC-DNA (Figure 3B), while those on C-DNA, 5mC-DNA, 5hmC-DNA and 5caC-DNA showed no obvious disparities before and after being reacted with PHPA under the same conditions (Figure 3C; Supplementary Figure S6a and b). The reduction in polymerization efficiency clearly arose from the specific labeling of 5fC with PHPA, because the three oxygen atoms in the sulfonic acid group may work as hydrogen-bonding acceptors that are capable of forming inter-molecular hydrogen bonds between the sulfonic acid group and amino acid residues in the polymerase. With increasing PHPA-5fC-DNA content (*m*_5fC_, mixed samples with diverse proportions of PHPA-5fC-DNA and DNA), the isothermal replication-scission amplification reaction showed a clear decrease in the polymerization efficiency of the KF polymerase. The Δ*S* increased linearly from 5.2 to 25.8% with an increase in the *m*_5fC_ from 10 to 100%, with a perfect fitting degree of the regression line of Δ*S* = 4.22*m*_5fC_ + 0.757 (*R*^2^ = 0.979, Figure 3D), indicating that the content of 5fC can be possibly achieved from Δ*S*.

Notably, the group introduced at epigenetic sites should both provide large steric hindrance and be water soluble. In addition to PHPA, biotin hydrazide was also examined for impeding the extension of DNA polymerase. However, no significant discrepancy was observed between the polymerization efficiency of the KF polymerase on 5fC-DNA and that on biotin-5fC-DNA, even when a huge complex was formed with streptavidin (streptavidin-biotin-5fC-DNA) (Supplementary Figure S7). This finding might be due to the extremely weak water solubility of biotin hydrazide, which likely resulted in its minimal reaction with 5fC-DNA.

**Figure 4.**
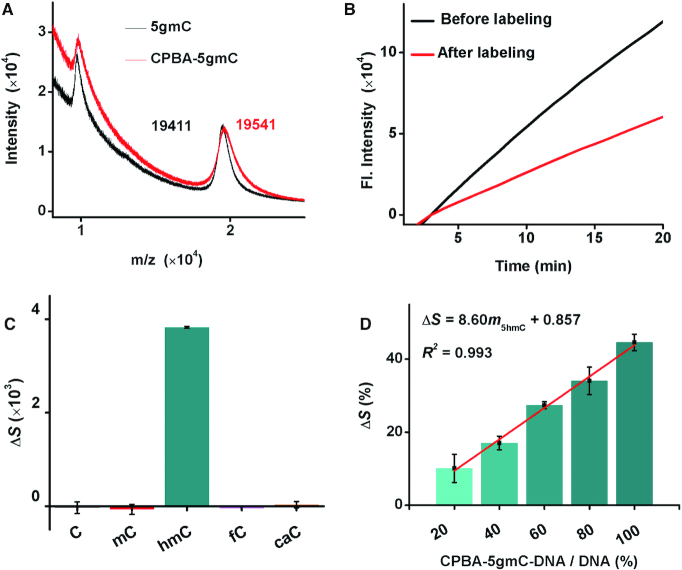
Feasibility of PRIA for the detection of 5hmC. (**A**) MALDI-TOF MS characterization of 5hmC-DNA after labeling with UDP-glucose and 3-CPBA. (**B**) Time-dependent fluorescence spectra of the isothermal replication-scission amplification reactions with 5hmC-DNA before and after labeling with UDP-glucose and 3-CPBA. (**C**) Histogram of Δ*S* for the isothermal replication-scission amplification reactions with C-DNA, 5mC-DNA, 5hmC-DNA, 5fC-DNA and 5caC-DNA before and after their reactions with T4-*β*GT/UDP-glucose and 3-CPBA. (**D**) Histogram of Δ*S* for the isothermal replication-scission amplification reactions with diverse proportions of CPBA-5gmC-DNA, and plot of Δ*S* versus CPBA-5gmC-DNA content.

Beyond 5fC, 5hmC can also be efficiently and specifically labeled with molecules, even though its hydroxyl is a low active group. Glucose is known to be transferred to the hydroxyl in 5hmC by T4-*β*GT and UDP-glucose specifically and efficiently, converting 5hmC to 5gmC. The *cis*-diol groups in the glucose moiety of 5gmC provides the possibility for addition of water-soluble boric acid derivatives through a mild cyclization reaction. Therefore, 5hmC-DNA was converted to CPBA-5gmC-DNA to improve the steric hindrance of 5hmC loci. As expected, via MALDI-TOF MS, a 5gmC-DNA signal with m/z 19411 was observed, and the chemical adduct CPBA-5gmC-DNA showed a signal with m/z 19541 (Figure 4A). After reacting with 3-CPBA, the *T*_m_ of double-stranded CPBA-5gmC-DNAs was reduced by 2.0°C compared to that of 5hmC-DNAs (Supplementary Figure S8 and Table S3). The labeling efficiency was evaluated by HPLC (monitored at 260 and 360 nm) and was ∼62.4% after the two-step reaction (Supplementary Figure S9 and Table S5). The labeling efficiency of 5hmC-DNAs with glucose/3-CPBA was also verified to be nearly constant among different samples (Supplementary Figure S10a–c). The polymerization efficiency of the KF polymerase showed a significant reduction (44.6%) on the chemical adduct CPBA-5gmC-DNA compared to that on 5hmC-DNA (Figure 4B). In contrast, little polymerization efficiency differences were found from amplification reactions on C-DNA, 5mC-DNA, 5fC-DNA and 5caC-DNA before and after being reacted with UDP-glucose, T4-*β*GT and 3-CPBA (Figure 4C and Supplementary Figure S11a–d). These results prove that the 3-CPBA is specific for 5gmC and greatly reduces the polymerization efficiency of DNA polymerase. The water solubility of 3-CPBA is increased by its carboxy group, as both hydrogen bond acceptors and donators in the carboxy can form hydrogen bonds with amino acid residues in the polymerase similar to PHPA-5fC-DNA. In addition, boric acid derivatives were reported to inhibit the extension of DNA polymerase (35). The amplification reaction showed a clear delay in the extension of the KF polymerase with diverse proportions of CPBA-5gmC-DNA, following a linear correlation of Δ*S* = 8.60*m*_5hmC_ + 0.857 (*R*^2^ = 0.993; Figure 4D).

**Figure 5.**
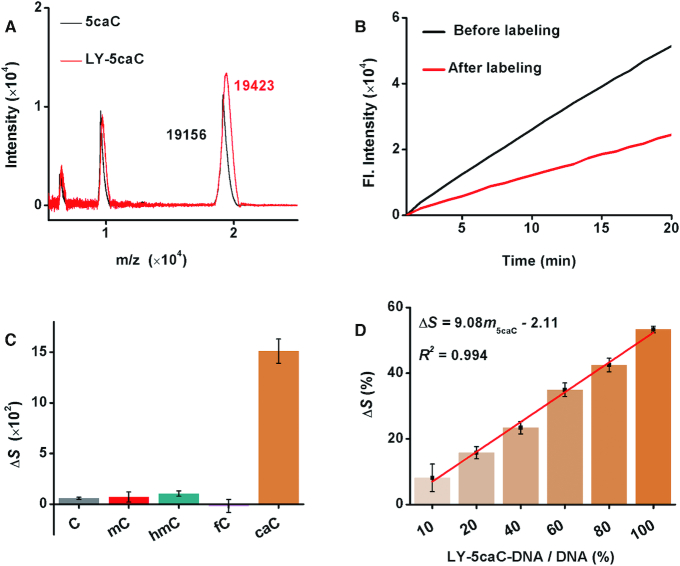
Feasibility of PRIA for the detection of 5caC. (**A**) MALDI-TOF MS of 5caC-DNA before and after labeling with LY. (**B**) Time-dependent fluorescence spectra of the isothermal replication-scission amplification reactions with 5caC-DNA before and after labeling with LY. (**C**) Histogram of Δ*S* for the isothermal replication-scission amplification reactions with C-DNA, 5mC-DNA, 5hmC-DNA, 5fC-DNA and 5caC-DNA before and after labeling with LY. (**D**) Histogram of Δ*S* for the isothermal replication-scission amplification reactions with diverse proportions of LY-5caC-DNA, and plot of Δ*S* versus LY-5caC-DNA content.

For 5caC, the carboxy in 5caC was exploited to react with an amine group or hydrazide, as amidation is a common condensation reaction in organic synthesis. The carboxyl in 5caC was activated by EDC combining with NHS, and being labeled with LY under mild conditions (Figure 1B). MALDI-TOF MS and LTQ-orbitrap-MS verified the signal of LY-5caC as expected (Figure 5A and Supplementary Figure S12a), and the *T*_m_ of double-stranded 5caC-DNAs was reduced by 1.0°C after being reacted with LY (Supplementary Figure S13 and Table S3). The labeling efficiency was evaluated by HPLC (monitored at 260 and 360 nm) and indicated that ∼51.9% of 5caC-DNA was converted into LY-5caC-DNA in the condensation reaction under mild conditions (Supplementary Figure S14 and Table S6). Neglectable discrepancy of labeling efficiency was found for different 5caC samples (Supplementary Figure S15a–c). LY-5caC resulted in a remarkable impeding effect on amplification efficiency. The polymerization efficiency of the amplification reaction was reduced by 53.3% on LY-5caC-DNA (Figure 5B), and the chemical modification reaction was highly specific, allowing 5caC to be differentiated from other bases specifically with striking discrepancy (Figure 5C and Supplementary Figure S16a–d). The LY-5caC-adduct also enabled quantification of the 5caC level, as the Δ*S* was positively relative to the content of LY-5caC, with a linear correlation of Δ*S* = 9.08*m*_5caC_ – 2.11 (*R*^2^ = 0.994; Figure 5D).

### Analytical precision and biological sample analysis

The reliability of the PRIA method was assessed via recovery experiments. Three artificially prepared DNA samples with different content of PHPA-5fC-DNA, ranging from 10 to 65 nM, were measured in parallel (*n* = 3). The results show recovery in the range of 97.2–101.1%. The parallel measurements of different concentrations of 5fC gave small relative standard deviations (RSDs) of 2.1–3.9% (Supplementary Table S7). The detection deviation and the possible nonspecific tagging of other bases in DNA samples might cause the recovery over 100%. However, compared to the previously reported works (36,37), the PRIA method has a good assay accuracy.

**Figure 6.**
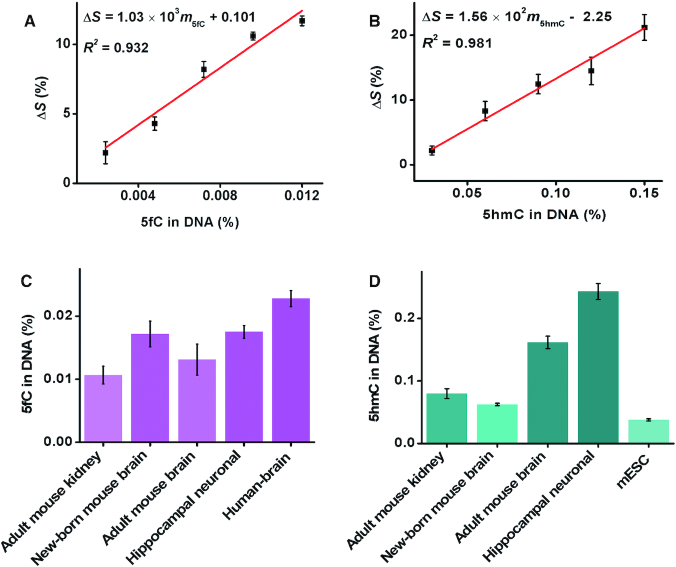
Biological sample analysis. (**A**) Quantitative calibration plots for 5fC in biological sample. (**B**) Quantitative calibration plots for 5hmC in biological sample. (**C**) Global 5fC levels in adult mouse kidney, new-born and adult mouse brain, rat hippocampal neuronal and human brain tissues. (**D**) Global 5hmC levels in adult mouse kidney, new-born and adult mouse brain, rat hippocampal neuronal tissues and mESCs.

The feasibility of the PRIA strategy for biological sample analysis was further investigated. A universal primer was designed to contain three parts similar to those of the model primer (5′-AGC CCG TGA GTC TCG GAC TGG AGT TCA GAC GTG TGC TCT TCC GAT C-3′). Genome-wide 5fC is rare and varies widely in different cells and tissues, but mESCs have relatively high levels of 5fC *in vivo*; thus, whole genomic dsDNA extracted from mESCs was used to construct a quantitative calibration curve for the detection of genomic 5fC. mESC dsDNA was sheared into small dsDNA fragments (about 200 bps) using ultrasound followed manufacturer's instructions (Supplementary Figure S17), and the dsDNA fragments were ligated to a universal tail (5′-GAC TGG AGT TCA GAC GTG TGC TCT TCC GAT C-3′) via NEBNext ultra end repair and dA-tailing and adaptor ligation. Under the optimal conditions (Supplementary Figure S18), amplification reactions with the ligated fragments (40 ng DNA) before and after their reaction with PHPA showed a clear polymerization efficiency difference, with an ∼11.7% reduction in the slope of the time-dependent fluorescence spectrum (Supplementary Figure S19). The decrease in the slope represents the global 5fC levels in mESC dsDNA (0.012% in DNA), which was quantified using a commercial MethylFlash™ 5-Formylcytosine DNA Quantification Kit (colorimetric) (Supplementary Figure S20). By analyzing the gradually diluted PHPA tagged mESC fragments, a quantitative calibration plot for 5fC was obtained. The Δ*S* increased linearly with the 5fC content in the range of 0.0024–0.012%, with a linear correlation of Δ*S* = 1.03 × 10^3^*m*_5fC_ + 0.101 (*R*^2^ = 0.932, Figure 6A). Similarly, a quantitative calibration plot for global 5hmC was obtained using adult human brain genomic DNA, since 5hmC content is relatively abundant in brain tissues. The slope of the time-dependent fluorescence spectrum was reduced by ∼21.2% with the T4-*β*GT/UDP-glucose and 3-CPBA-treated fragments (Supplementary Figure S21), which corresponds to the global level of 5hmC quantified by LC-MS/MS in adult human brain genomic DNA (0.15%) (Supplementary Figure S22a–c). The Δ*S* increased linearly with the 5hmC content in the range of 0.03–0.15%, with a linear correlation of Δ*S* = 1.56 × 10^2^*m*_5hmC_ − 2.25 (*R*^2^ = 0.981, Figure 6B). Notably, the quantitative calibration plot has the linear range covering the 5hmC levels found in most biological samples, and was established from the whole genomic sequence, thus is suitable for quantifying the 5hmC levels in different genomic DNAs of biological samples. Furthermore, the information of 5caC in mESCs genomic DNA was analyzed by PRIA strategy. A remarkable ∼23.1% reduction in the slope of the time-dependent fluorescence spectrum was observed (Supplementary Figure S23), which can be used to establish the quantitative calibration plot for 5caC (Supplementary Figure S24).

The applicability of the method to analyze 5fC in several biological samples including adult mouse kidney, new-born and adult mouse brain tissues, rat hippocampal neuronal tissue and human brain tissues. The detection results showed 5fC content of 0.0107, 0.0172, 0.0131, 0.0175 and 0.0228% per dN in DNA for adult mouse kidney, new-born and adult mouse brain tissues, rat hippocampal neuronal tissue and human brain tissues, respectively (Figure 6C). The results are in good agreement with ELISA kit estimation (Table 1). Besides 5fC, the information regarding 5hmC content was also quantified from adult mouse kidney, new-born and adult mouse brain tissues, rat hippocampal neuronal tissue and mESCs, and the levels of 5hmC were 0.0798, 0.0624, 0.161, 0.243 and 0.0378% per dN in DNA for adult mouse kidney, new-born and adult mouse brain tissues, rat hippocampal neuronal tissue and mESCs, respectively (Figure 6D). The results from PRIA are well consistent with those from the standard MS-based method (Table 2), indicating a good detection accuracy of the PRIA strategy. Based on the above data, much larger amount of 5hmC were observed in hippocampal neuronal tissue, which showed approximately 6 times as high as mESCs. In addition, relative high levels of 5hmC were also found in adult mouse brain tissue. The global levels of 5hmC increased age-dependent, exhibiting an inverted trend compared to 5fC. These variations between 5hmC and 5fC may link to active DNA demethylation, as 5fC were excised by Tdg protein with increasing age. This observations not only confirm the widespread presence of 5hmC and 5fC but also indicate their significant variation in different tissues and cells, which was in agreement with previous reports (2,8,18). It should be noted that 5-formyluracil (5fU) is potentially to react with PHPA. However, the available 5fU is originally extremely low and detectable amounts of 5fU are usually generated from exposure of DNA to *γ*-irradiation (38). Therefore, the rare 5fU in DNA would not make significant difference on detection of 5fC by PRIA.

**Table 1.** Estimation of 5fC in biological samples by ELISA and PRIA methods

	5fC in genomic DNA (%)		
Samples	Determined by ELISA	Determined by PRIA	Relative errors (%)
Adult mouse kidney	0.0133	0.0107	−19.5
New-born mouse brain	0.0194	0.0172	−11.3
Adult mouse brain	0.0134	0.0131	−2.2
Hippocampal neuronal	0.0172	0.0175	+1.7
Human brain	0.0239	0.0228	−4.6

**Table 2.** Estimation of 5hmC in biological samples by LC-MS/MS and PRIA methods

	5hmC in genomic DNA (%)		
Samples	Determined by LC-MS/MS	Determined by PRIA	Relative errors (%)
Adult mouse kidney	0.0702	0.0798	+12.0
New-born mouse brain	0.0617	0.0624	+1.1
Adult mouse brain	0.162	0.161	−0.6
Hippocampal neuronal	0.219	0.243	+10.3
mESC	0.0362	0.0378	+4.2

## DISCUSSION

As key epigenetic modifications, 5hmC, 5fC and 5caC are distributed in the genomic DNA of disparate tissues and cells (1−3). Determining the global levels of these 5mC-oxides are crucial for understanding their relationships and other biological functions. The current methods are generally costly, time-consuming, and require high sample input (Supplementary Table S8). The proposed PRIA strategy addresses these problems and shows some significant advantages as below:

- High sensitivity from novel amplification method. Conventional PCR-based methods, without bisulfite treatment, are unable to maintain epigenetic modification information after replication, moreover, the input DNA for bisulfite conversion is high (0.5–2 μg) (22,39,40). In comparison, the basic principle of our PRIA strategy is based on the fact that DNA polymerase inherently shows disparate polymerization efficiency when passing different nucleotides; therefore, information regarding modified loci are directly reserved during the replication process. By labeling distinct groups and forcing DNA polymerase to repetitively pass target oligo fragments, the PRIA strategy shows extremely high sensitivity such that extremely lowly abundant and even single 5mC-oxide nucleotides can be specifically and accurately distinguished based on the accumulated inefficiency of the polymerase.
- Rapid and facile with better assay accuracy. Instead of using harsh and burdensome bisulfite treatment or nucleoside digestion, the chemical labeling reactions involved in the PRIA strategy require no more than two steps and can be performed under mild conditions, greatly reduces the assay time and avoids the DNA damage. Moreover, thanks to the isothermal replication-scission amplification reaction of PRIA, which can be performed using an rt-qPCR instrument or microplate reader, multiple samples can be analyzed simultaneously in microplates (Supplementary Figure S25). And one biological sample with multiple modified cytosines can be analyzed by separate tagging and then comparing the polymerase timing of each modified sample to timing of the unmodified reference, which can also be facilely achieved on a 96-microplate.
- Cost-effective and low sample requirement. The PRIA assay does not depend on expensive, delicate instruments such as mass spectrometers and gene sequencers but instead needs only handy equipment such as an rt-qPCR instrument or microplate reader. The reagents for the chemical labeling reactions in the PRIA strategy are all common organic substances that are commercially available and less expensive than the antigen/antibody reagents used in ELISA assays. In addition, only nanoscale sample is required (100 ng DNA for initial ligation reaction and 40 ng DNA for PRIA assay), which is very important in epigenetic research as most of the clinical samples are limited and arduous to acquire.
- Good compatibility and wide expandability. The chemical labeling reactions are routine chemical reactions that can be performed in almost all research and medical laboratories. Moreover, polymerization efficiency was found to decrease with increasing numbers of *N*^6^-mA, which is a prevalent epigenetic modification in DNA and RNA with potentially important roles in biological functions (41,42). The methyl in the 6-amino group of *N*^6^-mA can disturb base pairing, likely due to the steric hindrance (33,34). We intend to further expand the application of PRIA to the analysis of *N*^6^-mA in real samples, facilitating the elucidation of the dynamic changes or potential epigenetic roles of *N*^6^-mA in mammals.

Although the PRIA shows high sensitivity and accuracy, the analytical performance of the method would be further improved. In order to avoid DNA damage, the labeling reactions in our work are performed under mild conditions, resulting in relatively low labeling efficiency. To perform the labeling reactions under a high temperature or a longer time, the conversion ratio of ‘tags’ to focal sites may be improved, and further improve the sensitivity and accuracy of detection. Besides, by integration with sequencing approaches, the application of the method is possibly extended to address more specific questions, such as to assess the relative abundance of these various modifications at specific genomic regions.

In summary, we propose a novel PRIA strategy capable of rapidly and sensitively quantifying the global levels of 5hmC, 5fC and 5caC in genomes without enrichment processes or bisulfite treatment. The strategy is reliable, cost-effective and only requires nanoscale sample input, and can accurately quantify 5hmC and 5fC in different biological samples using handy equipment in the laboratory. The strategy will likely be an appealing option for 5mC-oxide-focused studies in which numerous samples need to be analyzed quickly and inexpensively to gain biological insight, and is hopefully to advance the epigenetic researches.

## Supplementary Material

gkz704_Supplemental_File
