# Supplementary material for: Polymerization retardation isothermal amplification (PRIA): a strategy enables sensitively quantify genome-wide 5-methylcytosine oxides rapidly on handy instruments with nanoscale sample input
Source: Nucleic Acids Res. 2019 Aug 16;47(19):e119. doi: 10.1093/nar/gkz704 (PMC6821303; doi:10.1093/nar/gkz704)
Supplement: gkz704_Supplemental_File [file gkz704_supplemental_file.pdf]

Supporting Information

Polymerization Retardation Isothermal Amplification (PRIA): A strategy enables sensitively quantify genome-wide 5-methylcytosine oxides rapidly on handy instruments with nanoscale sample input

Danping Chen[1], Yang Wang[1], Mingming Mo[2], Junjie Zhang[2], Yanfei Zhang[1], Yuzhi Xu[1], Si-Yang Liu[1], Jun Chen[1], Yingjun Ma[1], Li Zhang[1], Zong Dai[1,*], Chun Cai[2] and Xiaoyong Zou[1]

1 School of Chemistry, Sun Yat-Sen University, Guangzhou 510275, China

2 Guangdong Key Laboratory for the Research and Development of Natural Drugs, Guangdong Medical College, Zhanjiang, Guangdong, 524023, China

Content

S1. Sequences of the used oligonucleotides....3
S2. Optimization of the isothermal replication-scission amplification reaction with template model....4
S3. Data processing....4
S4. Melting curves for double-stranded 5fC-DNAs before and after labeling with PHPA....5
S5. The T_m values of DNAs before and after chemical labeling....5
S6. Reaction specificity of PHPA verified by LTQ-orbitrap-MS....6
S7. Evaluation of labeling efficiency of PHPA for 5fC-DNA....8
S8. PRIA on DNAs before and after reaction with PHPA....10
S9. PRIA on biotin-5fC-DNA or streptavidin-biotin-5fC-DNA....11
S10. The T_m values of 5hmC-DNAs before and after chemical labeling....11
S11. Evaluation of labeling efficiency of CPBA for 5hmC-DNA....12
S12. PRIA on DNAs before and after reaction with 3-CPBA....14
S13. Reaction specificity of LY verified by LTQ-orbitrap-MS....15
S14. The T_m values of 5caC-DNAs before and after chemical labeling....17
S15. Evaluation of labeling efficiency of LY for 5caC-DNA....17
S16. PRIA on DNAs before and after reaction with LY....19
S17. Precision and recoveries of the PRIA strategy....19
S18. Pretreatment of genomic DNA....20
S19. Optimization of conditions for the isothermal replication-scission amplification reaction with genomic DNA....20
S20. Detection of 5fC in gemomic DNA....21
S21. Detection of 5fC in mESC by ELISA Kit....21
S22. Detection of 5hmC in genomic DNA....22
S23. Detection of 5hmC in adult human brain genomic DNA by LC-MS....23
S24. Detection of 5caC in genomic DNA....24
S25. Comparison of the methods for the analysis of epigenetic bases....25
S26. Detection of epigenetic bases in simultaneous way....26

S1. Sequences of the used oligonucleotides

Table S1 Sequence of the used oligos

Name	Sequence (5' → 3')
C-DNA	GAGACCGGAGTCCGCTTTCCTCTTCCGGAAAATGTAAGCCGAACCTAAAGCA ATCACCAGGG
5mC-DNA	GAGACCGGAGTCCGCTTTCCTCTTC**5mC**GGAAAATGTAAGCCGAACCTAAAG CAATCACCAGGG
5hmC-DNA-1	GAGACCGGAGTCCGCTTTCCTCTTC**5hmC**GGAAAATGTAAGCCGAACCTAAA GCAATCACCAGGG
5hmC-DNA-2	GAGAC**5hmC**GGAGTCCGCTTTCCTCTTCCGGAAAATGTAAGCCGAACCTAAA GCAATCACCAGGG
5hmC-DNA-3	GCCGAGAATGATTCAACCGGAGTTACTCTTC**5hmC**GGTCAGCCGAACCTAAA GCAATCACCAGGG
5fC-DNA	GAGACCGGAGTCCGCTTTCCTCTTC**5fC**GGAAAATGTAAGCCGAACCTAAAGC AATCACCAGGG
5caC-DNA-1	GAGACCGGAGTCCGCTTTCCTCTTC**5caC**GGAAAATGTAAGCCGAACCTAAAG CAATCACCAGGG
5caC-DNA-2	GAGAC**5caC**GGAGTCCGCTTTCCTCTTCCGGAAAATGTAAGCCGAACCTAAAG CAATCACCAGGG
5caC-DNA-3	GCCGAGAATGATTCAACCGGAGTTACTCTTC**5caC**GGTCAGCCGAACCTAAAG CAATCACCAGGG
Complementary-DNA	CCCTGGTGATTGCTTTAGGTTCGGCTTACATTTTCCGGAAGAGGAAAGCGGA CTCCGGTCTC
Primer-DNA	AGCCCGTGAGTCTCGCCCTGGTGATTGCTTTAGGTTCGGC
Primer-genome	AGCCCGTGAGTCTCGGACTGGAGTTCAGACGTGTGCTCTTCCGATC
A-RNA	GGACUGGACUGGACUGGACUGGACUAUCACCAGGG
mA1-RNA	GGACUGGACUGGACUGGACUGG**6mA**CUAUCACCAGGG
mA2-RNA	GGACUGGACUGGACUGG**6mA**CUGG**6mA**CUAUCACCAGGG
Primer-A	AGCCCGTGAGTCTCGCCCTGGTGATAGTCC
R_1	GTCGACCGGACGTCGAAA
R_2	GAACGGGATGAAGCCATTGCCAAGAAGCAGGCGGTGGAGGCGGAGCTGCA

S2. Optimization of the isothermal replication-scission amplification reaction with template model

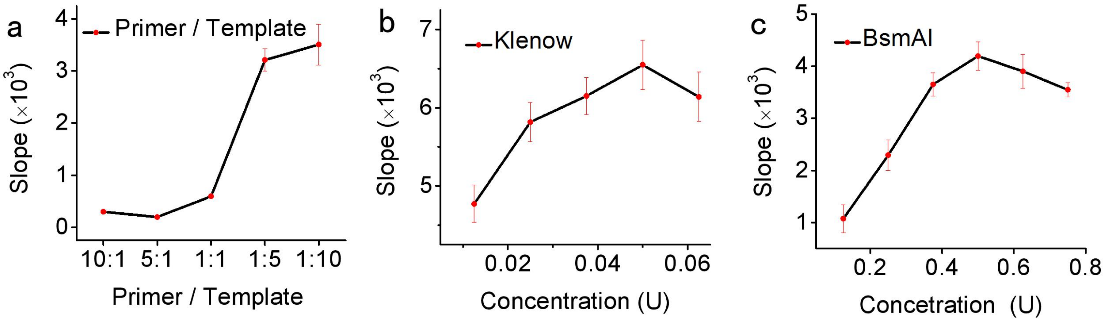

Figure S1. (a) Optimization of the molar ratio of the template and primer, the optimal molar ratio for primer to template was 1:5; (b) Optimization of the concentration of KF polymerase, the optimal concentration in the model reaction was 0.05 U; (c) Optimization of the concentration of nicking enzyme Nt.BsmAI, the optimal concentration in the model reaction was 0.5 U.

S3. Data processing

Table S2 Maximal slope screening in the confidence interval with R^2 > 0.9995

$Cycle_i$	$Cycle_j$	Slope	R^2
2	4	7410	0.9997
1	4	7165	~~0.9987~~
2	5	7283	0.9996
2	6	7234	0.9997
2	7	7216	0.9999
**2**	**8**	**7113**	**0.9995**
2	9	7024	~~0.9992~~
2	10	6952	~~0.9992~~

$Cycle_i$ presents the initial screening cycle site; $Cycle_j$ presents the terminal screening cycle site.

S4. Melting curves for double-stranded 5fC-DNAs before and after labeling with PHPA

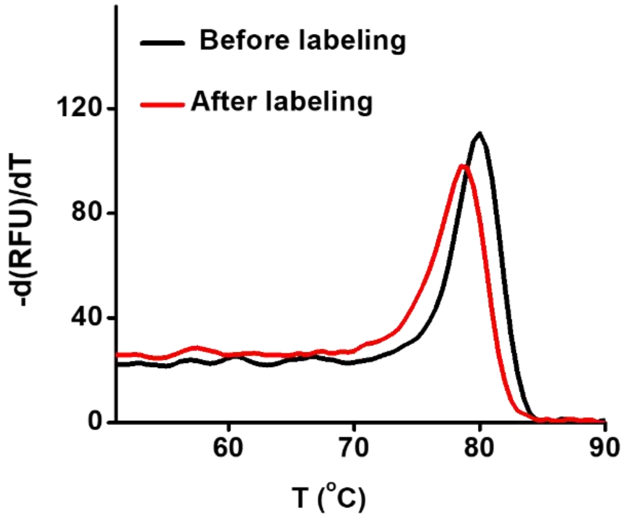

Figure S2. Melting curves for double-stranded 5fC-DNAs before and after labeling with PHPA.

S5. The T_m values of DNAs before and after chemical labeling

Table S3 T_m values of DNAs before and after chemical labeling

Sample	T_m (°C)		ΔT_m (°C)
	Before labeling	After labeling	
5fC-DNA	80.0	78.5	1.5
5hmC-DNAs	80.0	79[a], 78[b]	2.0
5caC-DNA	81.5	80.5	1.0

a, 5gmC; b, CPBA-5gmC.

S6. Reaction specificity of PHPA verified by LTQ-orbitrap-MS

a) PHPA reacted with 5fdC

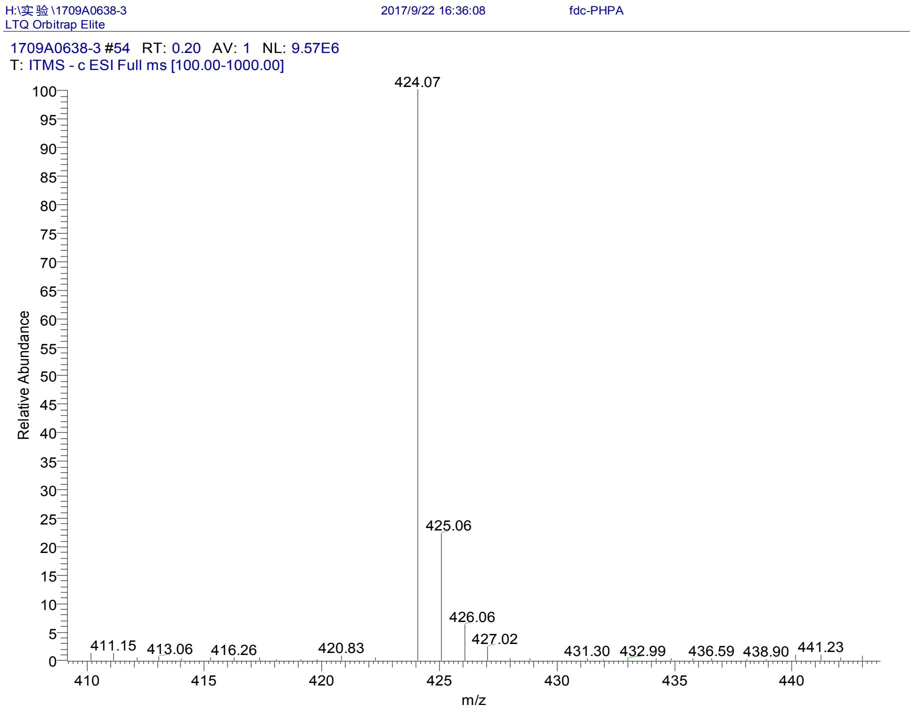

b) PHPA reacted with 5mdC

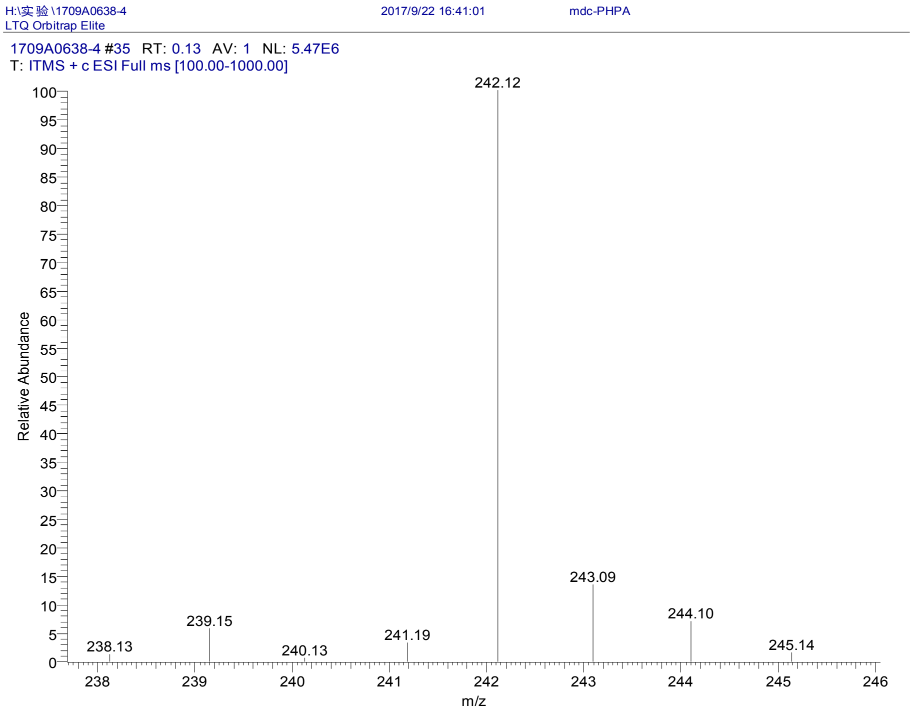

c) PHPA reacted with 5hmdC

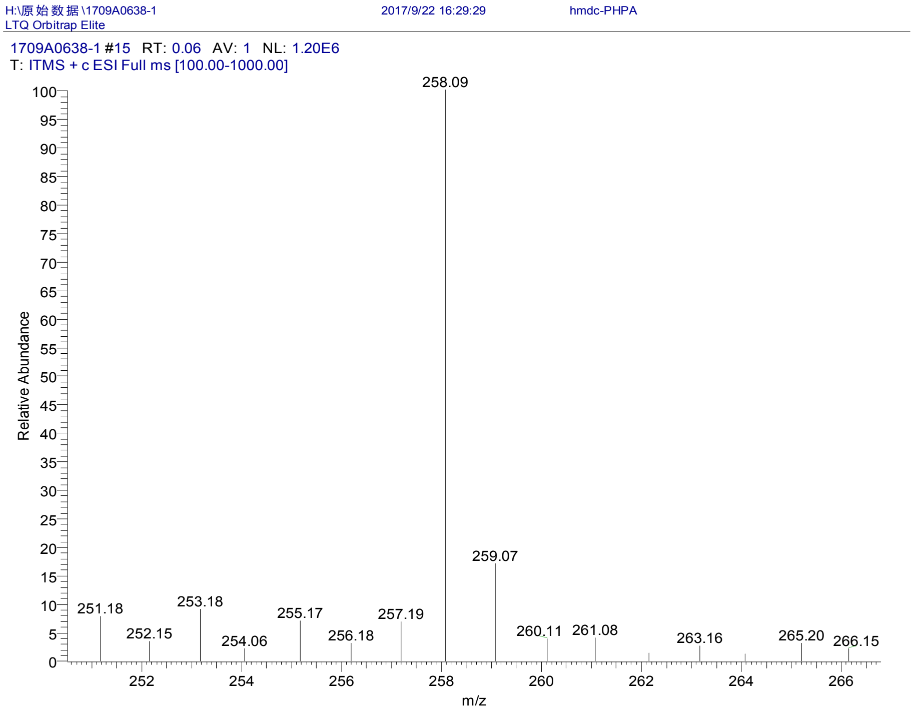

d) PHPA reacted with 5cadC

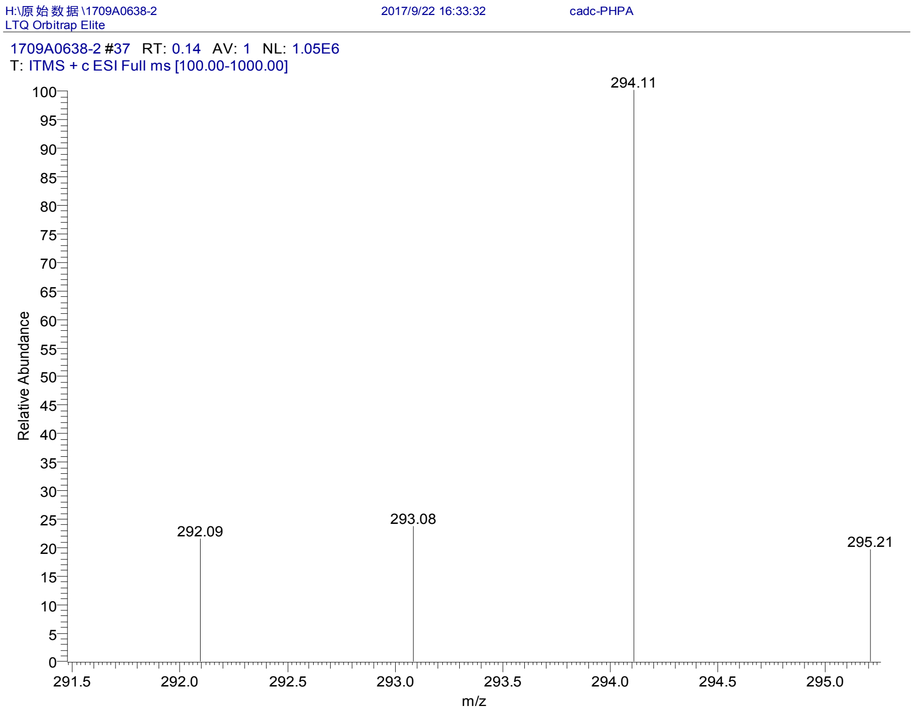

Figure S3. LTQ-orbitrap-MS analysis of the products between PHPA and (a) 5fdC, (b) 5mdC, (c) 5hmdC and (d) 5cadC, respectively.

S7. Evaluation of labeling efficiency of PHPA for 5fC-DNA

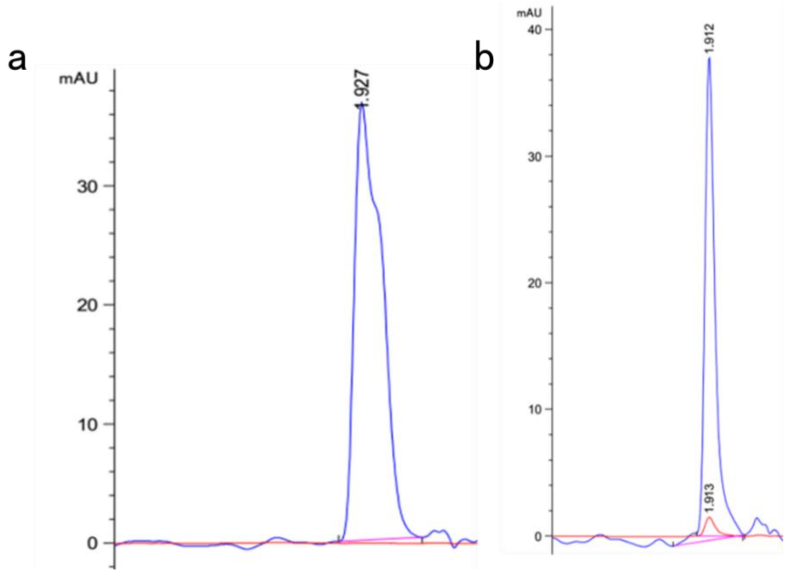

Figure S4. HPLC analysis of the conversion of (a) unlabeled 5fC-DNAs and (b) PHPA-5fC-DNAs.

Table S4 The conversion rate of PHPA-5fC from 5fC

Peak	Ret time (min)	Width (min)	Area (mAU×s)	Hight (mAU)	Conversion (%)
5fC	1.927	0.1996	534.4	36.77	64.77
PHPA-5fC	1.912	0.1339	346.1	38.12	

Ret Time, Retention time.

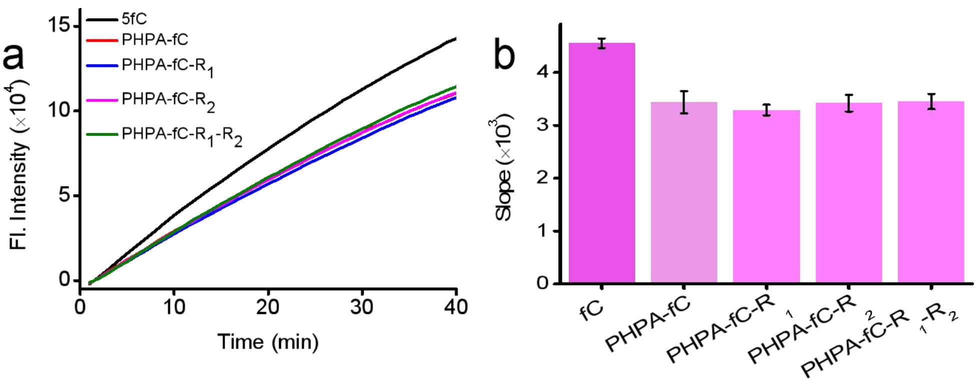

Figure S5. Real-time fluorescence curves (a) and histogram graph (b) of 5fC, PHPA-fC, mixture of PHPA-fC-R_1 (PHPA-fC-R_1 represents PHPA-fC blends with short strand R_1), mixture of PHPA-fC-R_2 (PHPA-fC-R_2 represents PHPA-fC blends with long strand R_2), mixture of PHPA-fC-R_1-R_2, respectively.

S8. PRIA on DNAs before and after reaction with PHPA

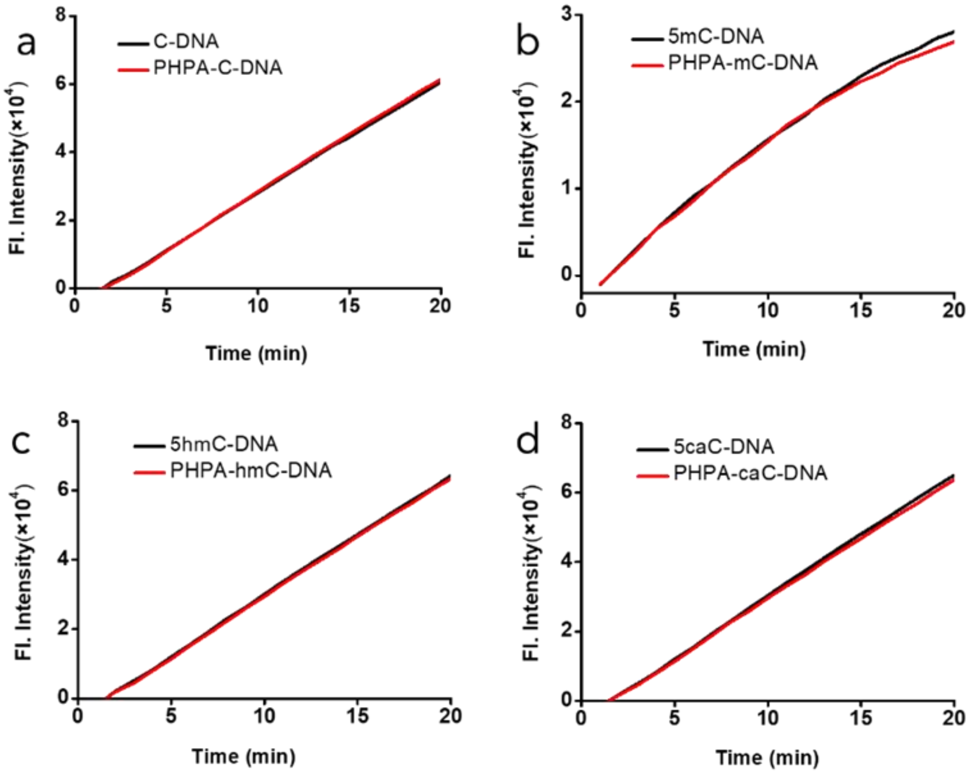

Figure S6. Time-dependent fluorescence spectra of the isothermal replication-scission amplification reactions with (a) C-DNAs, (b) 5mC-DNAs, (c) 5hmC-DNAs and (d) 5caC-DNAs before and after reaction with PHPA, respectively.

S9. PRIA on biotin-5fC-DNA or streptavidin-biotin-5fC-DNA

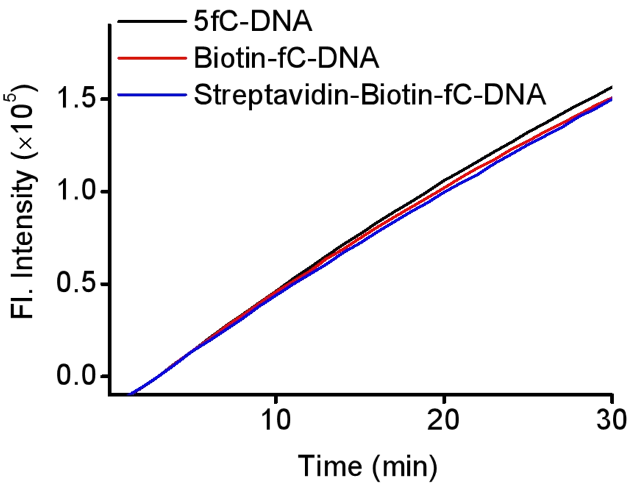

Figure S7. Time-dependent fluorescence spectra of the isothermal replication-scission amplification reactions with 5fC-DNA, Biotin-5fC-DNA and Streptavidin-Biotin-5fC-DNA respectively.

S10. The T_m values of 5hmC-DNAs before and after chemical labeling

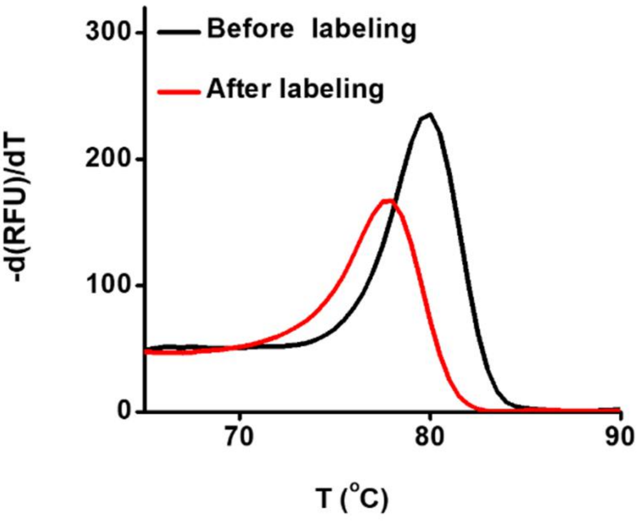

Figure S8. Melting curves for double-stranded 5hmC-DNAs before and after labeling with T4-βGT/UDP-glucose and 3-CPBA.

S11. Evaluation of labeling efficiency of CPBA for 5hmC-DNA

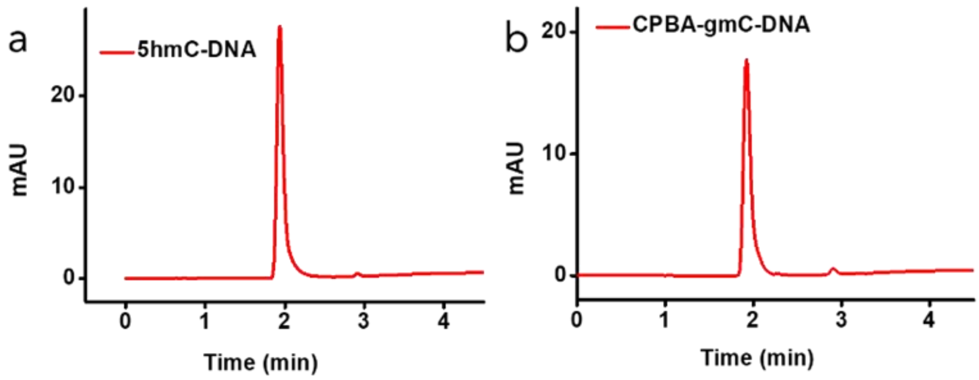

Figure S9. HPLC analysis of the (a) unlabeled 5hmC-DNAs and (b) CPBA-gmC-DNAs.

Table S5 The conversion rate of CPBA-gmC from 5hmC

Peak	Ret time (min)	Width (min)	Area (mAU×s)	Hight (mAU)	Conversion (%)
5hmC	1.936	0.09430	175.5	27.55	62.36
CPBA-5gmC	1.923	0.09420	109.5	17.74	

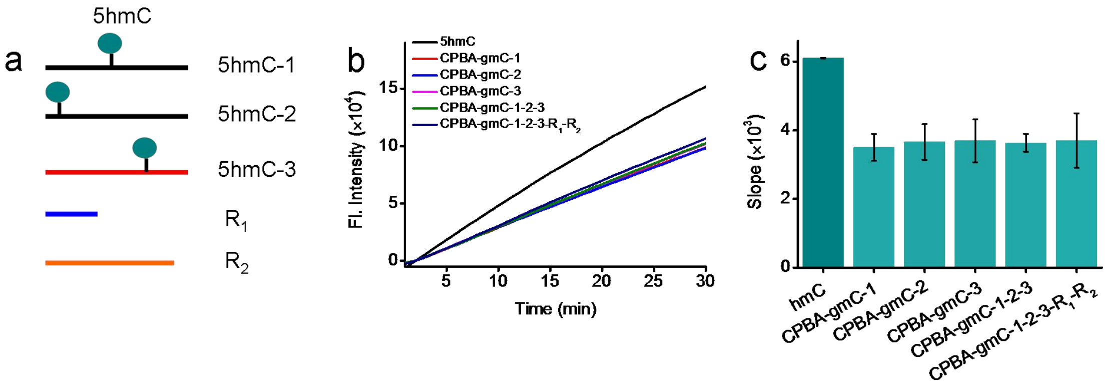

Figure S10. (a) Different types of DNA strands (the black lines represent DNA strand with the same sequence but different 5hmC sites, the red line and black line represent DNA strand with different sequence). Real-time fluorescence curves (b) and histogram graph (c) of 5hmC; CPBA-gmC-1, CPBA-gmC-2, CPBA-gmC-3, the mixture of CPBA-gmC-1-2-3 (CPBA-gmC-1-2-3 represents CPBA-gmC-1 blends with CPBA-gmC-2 and CPBA-gmC-3; the mixture contains the sames amount of focal sites with other samples, 100 nM), mixture of CPBA-gmC-1-2-3-R_1-R_2, respectively.

S12. PRIA on DNAs before and after reaction with 3-CPBA

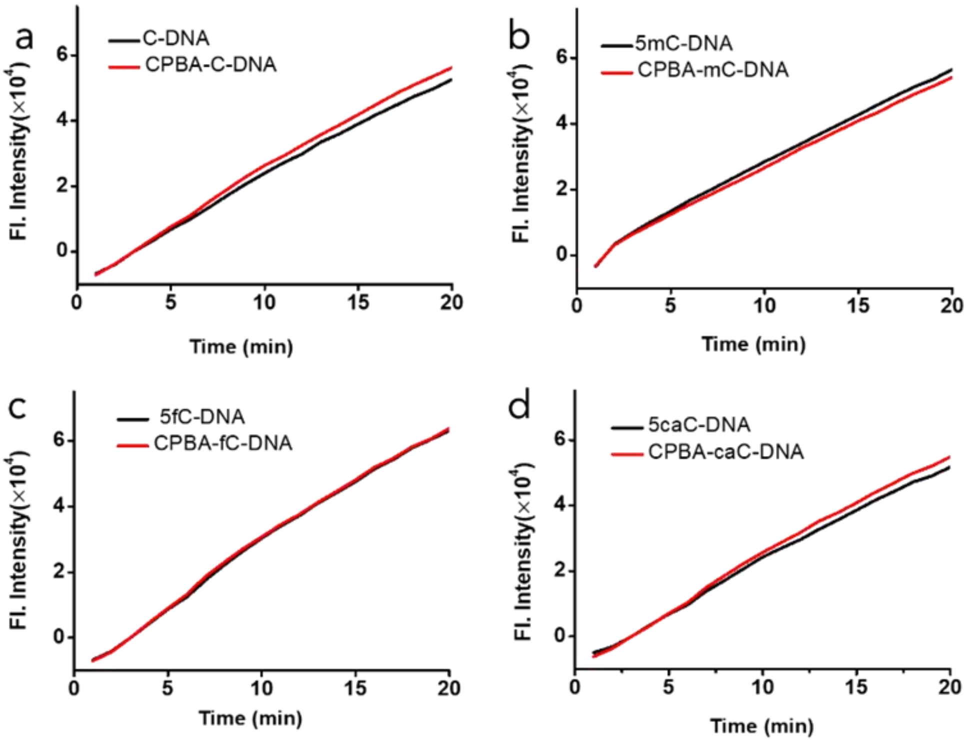

Figure S11. Time-dependent fluorescence spectra of the isothermal replication-scission amplification reactions with (a) C-DNAs, (b) 5mC-DNAs, (c) 5fC-DNAs and (d) 5caC-DNAs before and after reaction with CPBA, respectively.

S13. Reaction specificity of LY verified by LTQ-orbitrap-MS

a) LY reacted with 5cadC

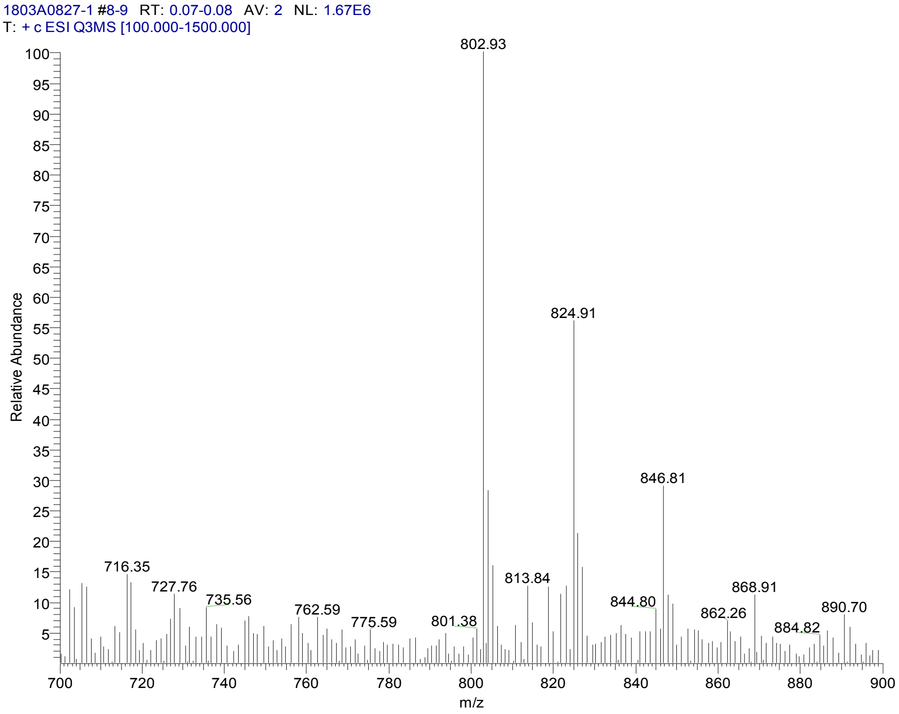

b) LY reacted with 5mdC

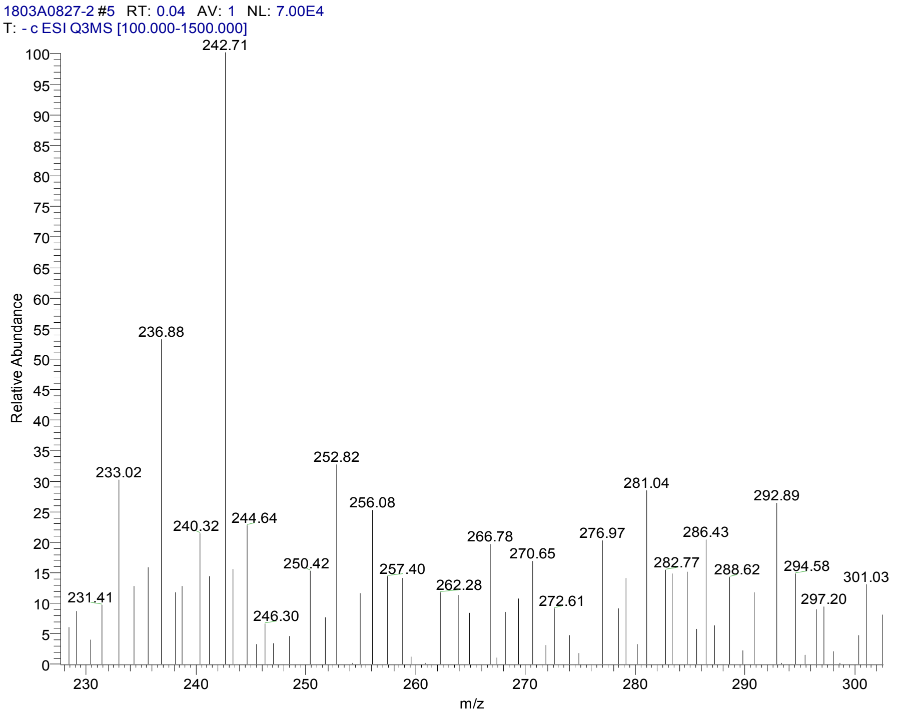

c) LY reacted with 5hmdC

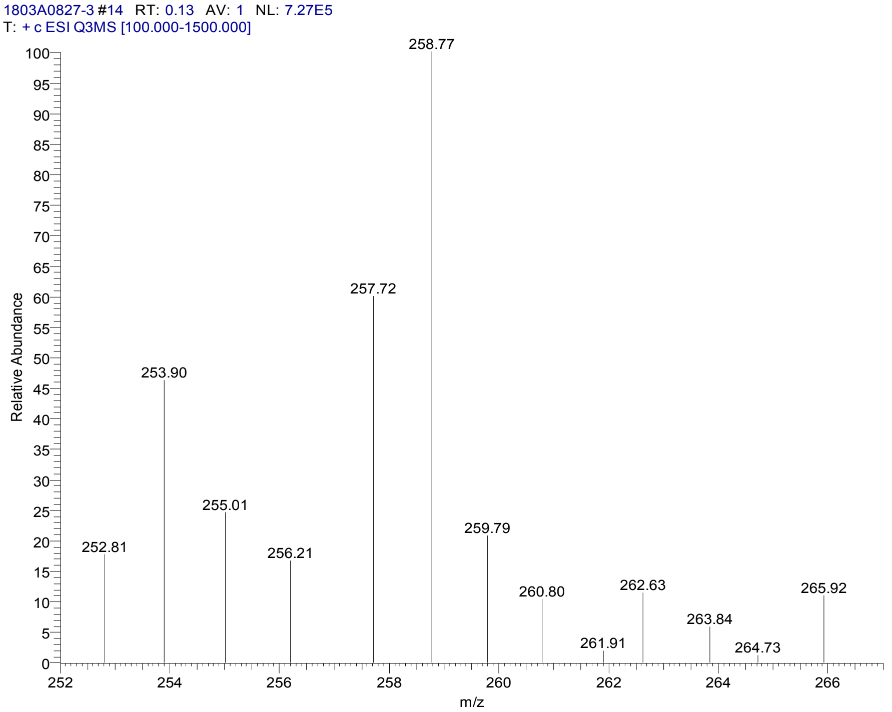

d) LY reacted with 5fdC

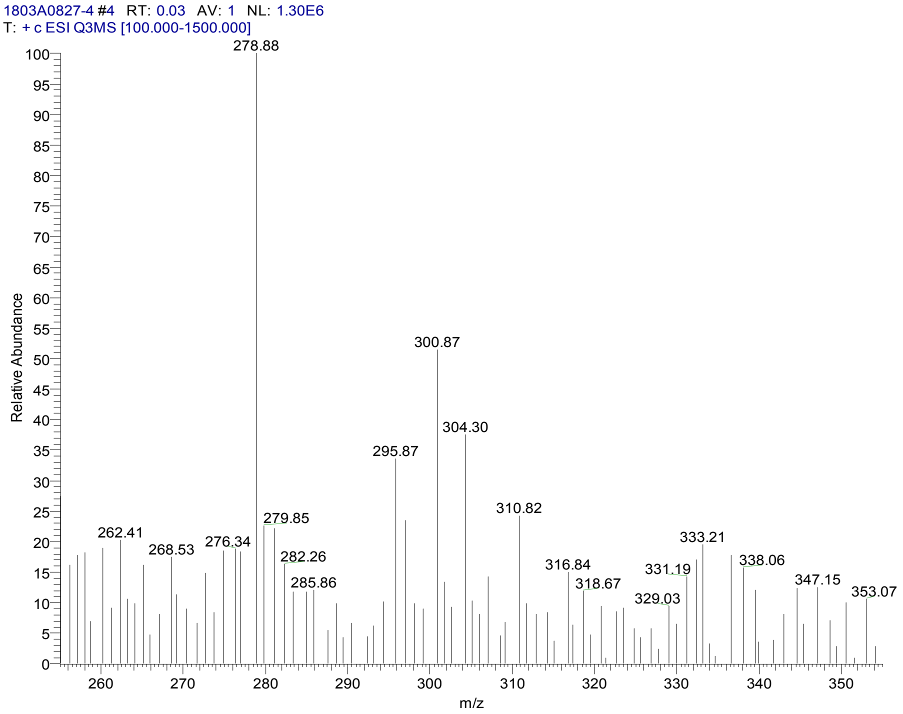

Figure S12. LTQ-orbitrap-MS analysis of the product between LY and (a) 5cadC, (b) 5mdC, (c) 5hmdC and (d) 5fdC, respectively.

S14. The T_m values of 5caC-DNAs before and after chemical labeling

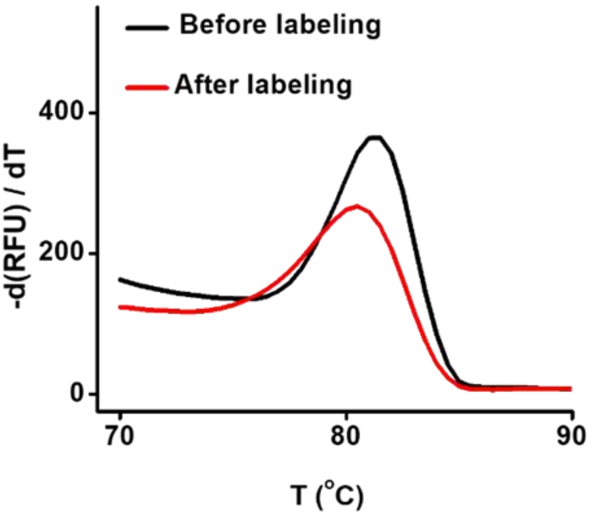

Figure S13. Melting curves for double-stranded 5caC-DNAs before and after labeling with LY.

S15. Evaluation of labeling efficiency of LY for 5caC-DNA

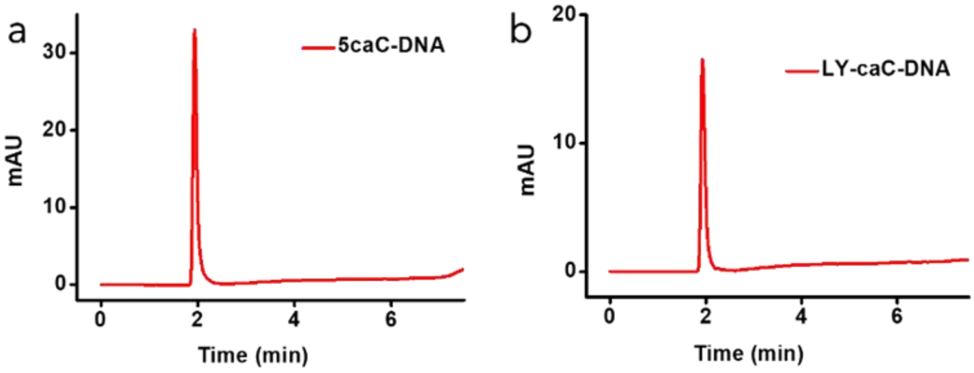

Figure S14. HPLC analysis of the conversion of (a) unlabeled 5caC-DNAs and (b) LY-5caC-DNAs.

Table S6 The conversion rate of LY-5caC from 5caC

Peak	Ret time (min)	Width (min)	Area (mAU×s)	Hight (mAU)	Conversion (%)
5caC	1.934	0.09560	208.8	33.11	51.93
LY-5caC	1931	0.09860	108.4	16.52	

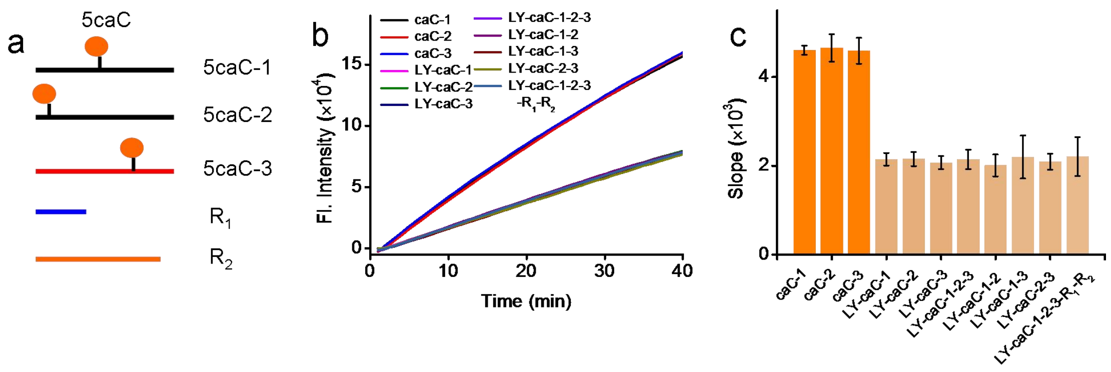

Figure S15. (a) Different types of DNA strands (the black lines represent DNA strands with the same sequence but different 5caC sites, the red line and black line represent DNA strands with different sequence). Real-time fluorescence curves (b) and histogram graph (c) of different 5caC-1, 5caC-2, 5caC-3; LY-caC-1, LY-caC-2, LY-caC-3, mixture of LY-caC-1-2-3 (LY-caC-1-2-3 represents LY-caC-1 blends with LY-caC-2 and LY-caC-3; the mixture contains the sames amount of focal sites with other samples, 100 nM), mixture of LY-caC-1-2, mixture of LY-caC-1-3, mixture of LY-caC-2-3, mixture of LY-caC-1-2-3-R_1-R_2, respectively.

S16. PRIA on DNAs before and after reaction with LY

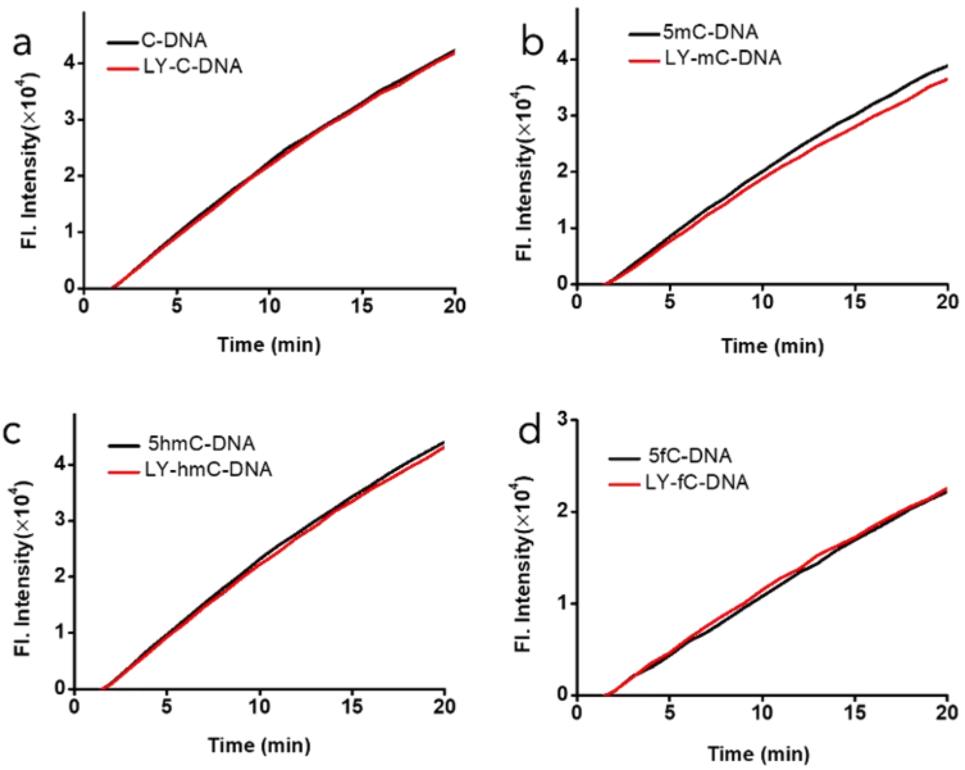

Figure S16. Time-dependent fluorescence spectra of the isothermal replication-scission amplification reactions with (a) C-DNAs, (b) 5mC-DNAs, (c) 5hmC-DNAs and (d) 5fC-DNAs before and after reaction with LY, respectively.

S17. Precision and recoveries of the PRIA strategy

Table S7 Precision and recoveries for the determination of 5fC by PRIA

Sample	Detected (nM)	Added (nM)	Found (nM)	Recovery (%)	R.S.D (%)
Sample 1	4.90	10.0	14.9	100.0	3.7
Sample 2	4.90	25.0	29.2	97.2	2.1
Sample 3	4.90	65.0	70.6	101.1	3.9

S18. Pretreatment of genomic DNA

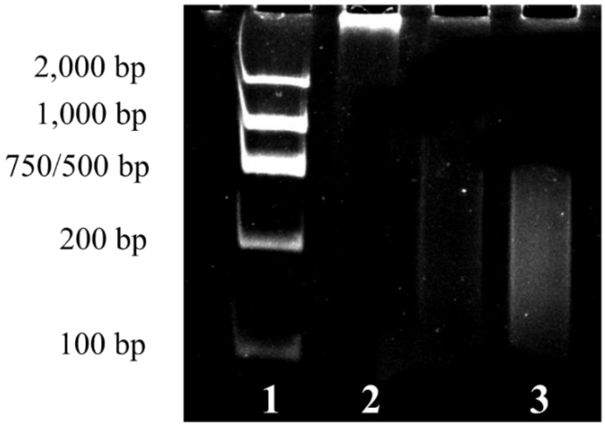

Figure S17. PAGE analysis of the fragmentation of whole genome DNA. Lane 1 was DNA marker, lane 2 was genomic DNA, and lane 3 was approximately 200 bp fragments after ultrasonic shearing of adult human brain genomic DNA.

S19. Optimization of conditions for the isothermal replication-scission amplification reaction with genomic DNA.

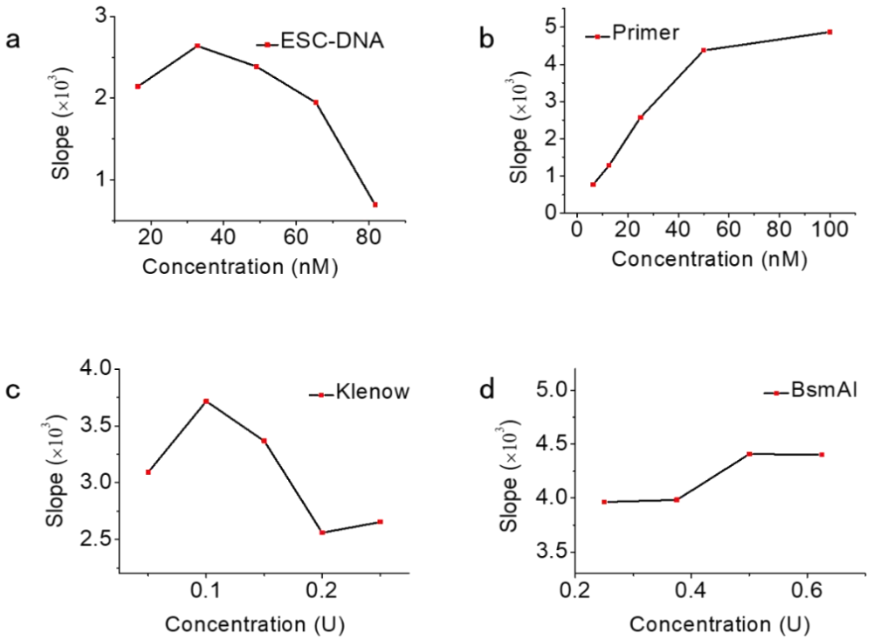

Figure S18. Optimization of conditions for the isothermal replication-scission amplification reaction with genomic DNA. (a) genomic template; (b) primer content; (c) concentration of KF; (d) concentration of Nt.BsmAI in genomic amplification reaction. The optimal contents of template and primer were 32.8 nM (about 40 ng DNA), 50 nM respectively, and the optimal concentrations of KF and Nt.BsmAI were 0.1 U and 0.5 U respectively.

S20. Detection of 5fC in genomic DNA

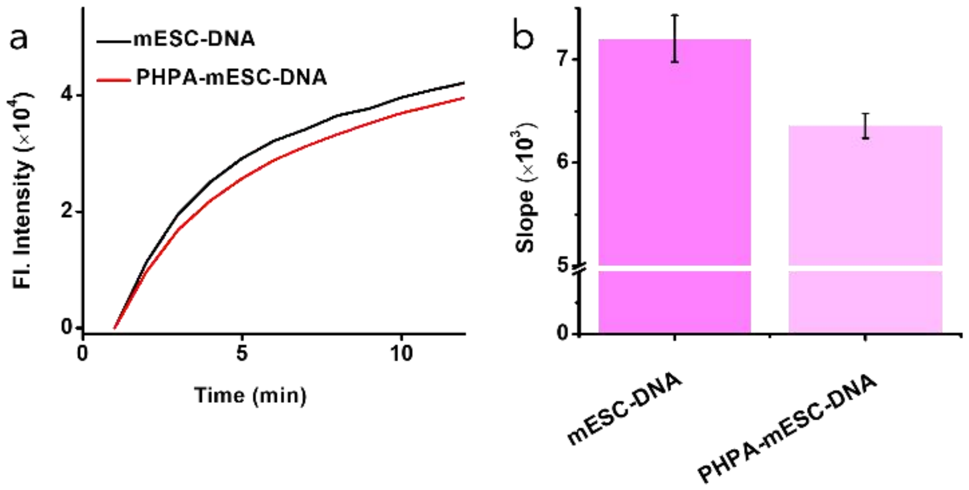

Figure S19. (a) Time-dependent fluorescence spectra of the isothermal replication-scission amplification reactions with the DNAs extracted from the mESC (control) and after labeling with PHPA (PHPA-mESC-DNA). (b) Histogram of the isothermal replication-scission amplification reaction slope with mESC-DNAs before and after labeling with PHPA. The slope was found reduced by 11.7%.

S21. Detection of 5fC in mESC by ELISA Kit

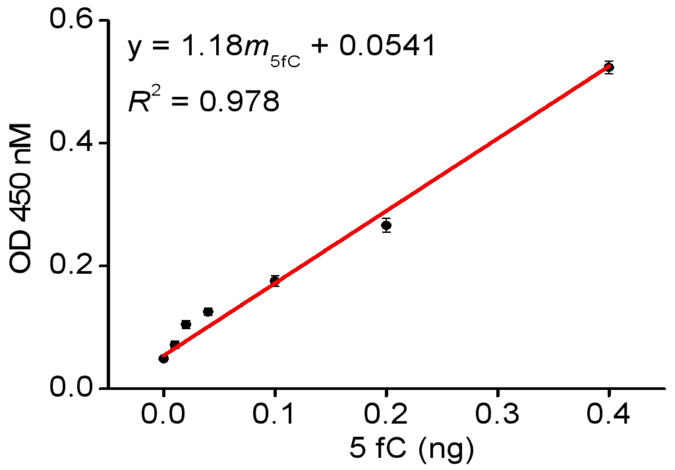

Figure S20. Calibration plot obtained by ELISA kit for quantitation of the 5fC extracted from mESC. The content of 5fC was 0.012% in DNA based on this calibration plot.

S22. Detection of 5hmC in genomic DNA

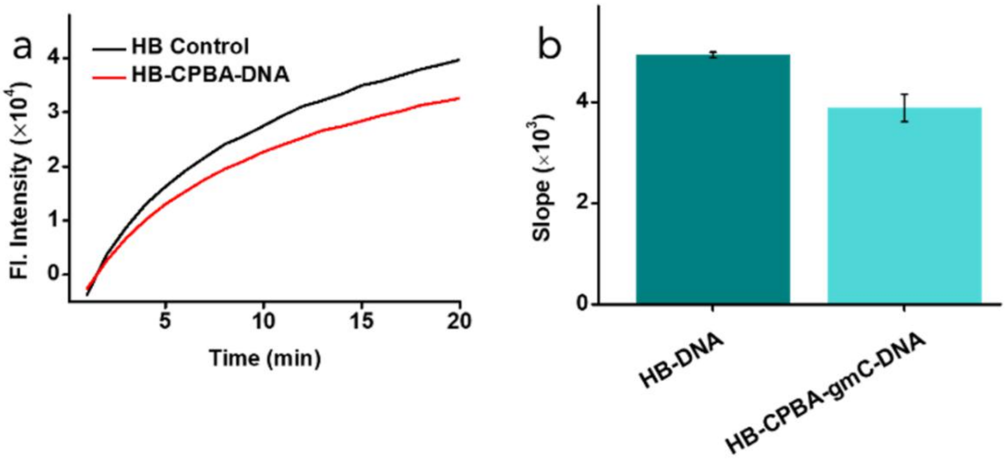

Figure S21. (a) Time-dependent fluorescence spectra of the isothermal replication-scission amplification reactions with the DNAs extracted from the adult human brain tissue before (control) and after labeling with 3-CPBA (HB-CPBA-gmC). (b) Histogram of the isothermal replication-scission amplification reaction slope with HB DNAs before and after labeling with 3-CPBA. The slope was found reduced by 21.2%.

S23. Detection of 5hmC in adult human brain genomic DNA by LC-MS

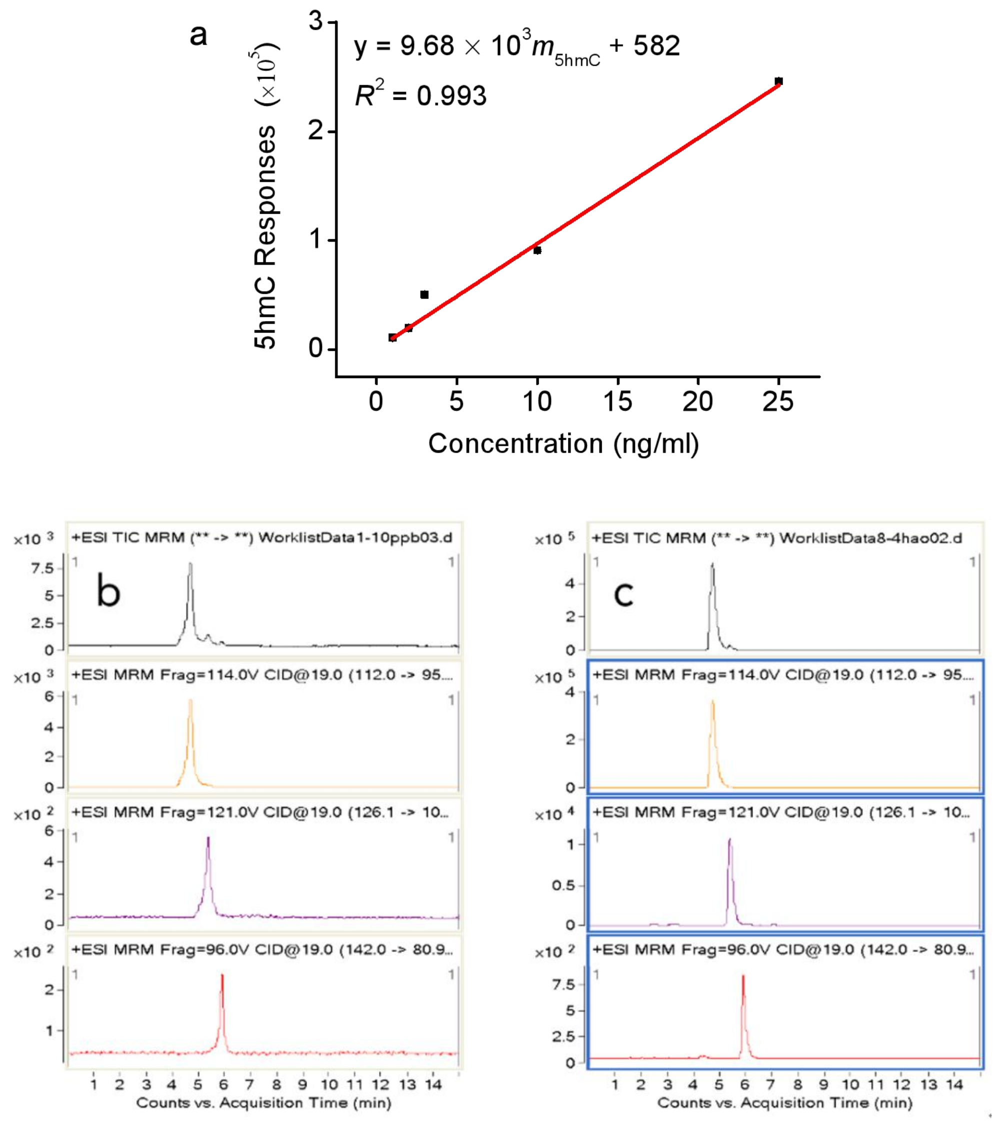

Figure S22. (a) Calibration plot obtained by LC-MS/MS for quantitation of the total content of 5hmC in human brain genomic DNA; (b) Mass chromatograms of the quantifer transition for 5hmC standards; (c) Mass chromatograms of the quantifer transition for adult human brain genomic DNA. The content of 5hmC was quantified to be 0.15% in DNA.

S24. Detection of 5caC in genomic DNA

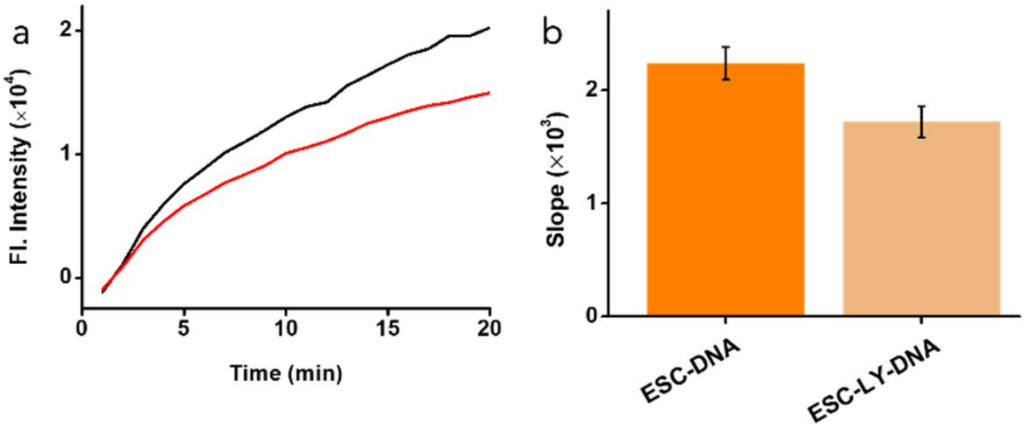

Figure S23. (a) Time-dependent fluorescence spectra of the isothermal replication-scission amplification reactions with the DNAs extracted from the mESC before (ESC-caC) and after labeling with LY (ESC-LY-5caC); (b) Histogram of the isothermal replication-scission amplification reaction slope with mESC DNAs before and after labeling with LY. The slope was found reduced by 23.1%.

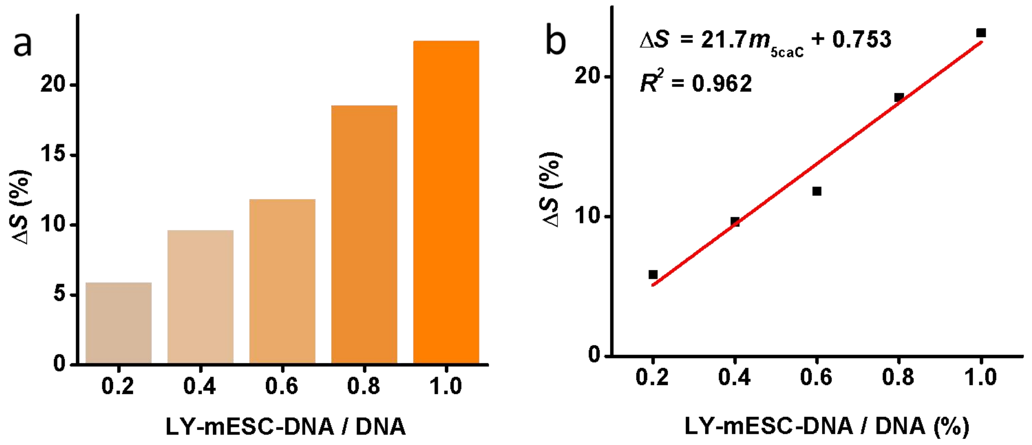

Figure S24. (a) Histogram of Δ*S* for isothermal replication-scission amplification reactions with diverse proportions of LY-5caC genomic DNA; (b) Plot between ΔS and the 5caC-DNA content.

S25. Comparison of the methods for the analysis of epigenetic bases

Table S8 Comparison of the methods for the analysis of epigenetic bases

Methods	Category	Analyte	Sample input	Pretreatment	Cost	Instrument	Time /h	Ref
MS-based	LC-MS/MS<br>HPLC-ESI MS/MS/MS	5mC<br>5hmC, 5fC, 5caC	1-4 μg	Nucleoside digestion, Enrichment	High	MS	> 24	1-5
BS-seq	oxBS-seq<br>redBS-seq	5hmC<br>5fC	1-3 μg	Bisulfite	High	Sequencers	40-50	6,7
PCR		5mC	0.5-2 μg	Bisulfite	High	qPCR	> 20	8,9
ELISA		5mC, 5hmC, 5fC,	1-4 μg	Antigen/Antibody	Medium	Microplate reader	> 20	10-12
TLC		5mC, 5hmC, 5fC, 5caC,	1-2 μg	Nucleoside digestion, Isotopic labelling	Low	PhosphorImager	> 10	13-14
PRIA		5hmC, 5fC, 5caC	100 ng	Adaptor ligation	Low	Thermostatic apparatus	< 10	This work

S26. Detection of epigenetic bases in simultaneous way.

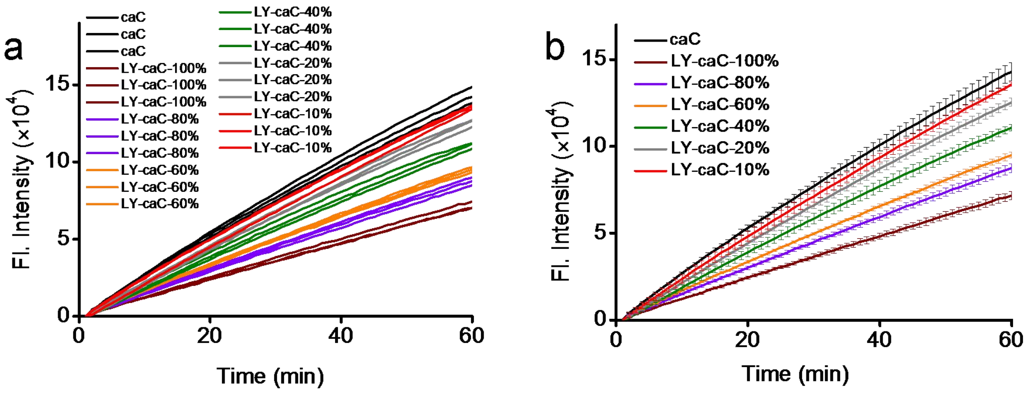

Figure S25. (a) Time-dependent fluorescence spectra of the isothermal replication-scission amplification reactions with diverse proportions of LY-5caC and 5caC (the LY-5caC contents were 0%, 10%, 20%, 40%, 60%, 80% and 100% in the mixture of 5caC and LY-5caC) in simultaneously; (b) The average response of the fluorescence signals from same LY-caC content.

References

1. Zhang, L. T., Zhang, L. J., Zhang, J. J., Ye, X. X., Xie, A. M., Kang, J. X. and Cai, C. (2013) Quantification of the sixth DNA base 5-hydroxymethylcytosine in colorectal cancer tissue and C-26 cell line, *Bioanalysis*, **5**, 839-845.
2. Zhang, L., Szulwach, K. E., Hon, G. C., Song, C. X., Park, B., Yu, M., Lu, X. Y., Dai, Q., Wang, X., Street, C. R., Tan, H. P., Min, J. H., Ren, B., Jin, P. and He, C. (2013) Tet-mediated covalent labelling of 5-methylcytosine for its genome-wide detection and sequencing, *Nat. Commun.*, **4**, 1517.
3. Münzel, M., Globisch, D., Brückl, T., Wagner, M., Michalakis, S., Welzmiller, V., Michalakis, S., Müller, M., Biel, M. and Carell, T., (2010) Quantification of the sixth DNA base hydroxymethylcytosine in the brain, *Angew. Chem. Int. Ed.*, **49**, 5375-5377.
4. Pfaffeneder, T., Hackner, B., Truβ, H. M., Münzel, M., Müller, M., Deiml, C. A., Hagemeier, C. and Carell, T. (2011) The discovery of 5-formylcytosine in embryonic stem cell DNA, *Angew. Chem. Int. Ed.*, **50**, 7008-7012.
5. Liu, S., Wang, J., Su, Y. J., Guerrero, C., Zeng, Y. X., Mitra, D., Brooks, P. J., Fisher, D. E., Song, H. J. and Wang, Y. S. (2013) Quantitative assessment of Tet-induced oxidation products of 5-methylcytosine in cellular and tissue DNA, *Nucleic Acids Res.*, **41**, 6421-6429.
6. Booth, M. J., Marsico, G., Bachman, M., Beraldi, D. and Balasubramanian, S. (2014) Quantitative sequencing of 5-formylcytosine in DNA at single-base resolution, *Nat. Chem*., **6**, 435-440.
7. Booth, M. J., Branco, M. R., Ficz, G., Oxley, D., Krueger, F., Reik, W. and Balasubramanian, S. (2012) Quantitative sequencing of 5-methylcytosine and 5-hydroxymethylcytosine at single-base resolution, *Science*, **336**, 934-937.
8. Huang, Y., Pastor, W. A., Shen, Y. H., Tahiliani, M, Liu, D. R and Rao, A. (2010) The behaviour of 5-hydroxymethylcytosine in bisulfite sequencing, *PLoS ONE*, **5**, e8888.
9. Wang, S. R., Chen, Y. Q., Hong, T. T., He, Z. Y., Guo, S., Lai, H., Guo, G., Du, Y. H. and Zou, Y. (2016) Simultaneous and sensitive detection of multisite 5-methylcytosine including non-CpG sites at single-5mC-resolution, *Anal. Chem.*, **88**, 10547-10551.
10. Ito, S., Shen, L., Dai, Q., Wu, S. C., Collins, L. B., Swenberg, J. A., He, C. and Zhang, Y. (2011) Tet proteins can convert 5-methylcytosine to 5-formylcytosine and 5-carboxylcytosine, *Science*, **333**, 1300-1303.
11. Szulwach, K. E., Li, X. K., Smrt, R. D., Li, Y. J., Luo, Y. P., Lin, L., Santistevan, N. J., Li, W., Zhao, X. Y. and Jin, P. (2010) Cross talk between microRNA and epigenetic

regulation in adult neurogenesis, *J. Cell. Biol.*, **189**, 127-141.
12. Weber, M., Davies, J. J., Wittig, D., Oakeley, E. J., Haase, M., Lam, W. L. and Schübeler, D. (2005) Chromosome-wide and promoter-specific analyses identify sites of differential DNA methylation in normal and transformed human cells, *Nat. Genet.*, **37**, 853-862.
13. He, Y. F., Li, B. Z., Li, Z., Liu, P., Wang, Y., Tang, Q., Ding, J., Jia, Y., Chen, Z., Li, L., Sun, Y., Li, X., Dai, Q., Song, C. X., Zhang, K., He, C. and Xu, G. L. (2011) Tet-mediated formation of 5-carboxylcytosine and its excision by TDG in mammalian DNA, *Science*, **333**, 1303-1307.
14. Tahiliani, M., Koh, K. P.,Shen, Y. H., Pastor, W. A., Bandukwala, H., Brudno, Y., Agarwal, S., Lyer, L. M., Liu, D. R., Aravind, L. and Rao, A. (2009) Conversion of 5-methylcytosine to 5-hydroxymethylcytosine in mammalian DNA by MLL partner TET1, *Science*, **324**, 930-935.
